# Contrasting Effects of the Cytotoxic Anticancer Drug Gemcitabine and the EGFR Tyrosine Kinase Inhibitor Gefitinib on NK Cell-Mediated Cytotoxicity via Regulation of NKG2D Ligand in Non-Small-Cell Lung Cancer Cells

**DOI:** 10.1371/journal.pone.0139809

**Published:** 2015-10-06

**Authors:** Riki Okita, Diana Wolf, Koichiro Yasuda, Ai Maeda, Takuro Yukawa, Shinsuke Saisho, Katsuhiko Shimizu, Yoshiyuki Yamaguchi, Mikio Oka, Eiichi Nakayama, Andreas Lundqvist, Rolf Kiessling, Barbara Seliger, Masao Nakata

**Affiliations:** 1 Department of General Thoracic Surgery, Kawasaki Medical School, Kurashiki, Japan; 2 Department of Clinical Oncology, Kawasaki Medical School, Kurashiki, Japan; 3 Department of Respiratory Medicine, Kawasaki Medical School, Kurashiki, Japan; 4 Faculty of Health and Welfare, Kawasaki University of Medical Welfare, Kurashiki, Japan; 5 Department of Oncology and Pathology, Immune and Gene Therapy Laboratory, Cancer Center Karolinska, Karolinska Institutet, Stockholm, Sweden; 6 Institute of Medical Immunology, Martin Luther University, Halle-Wittenberg, Halle, Germany; Winship Cancer Institute of Emory University, UNITED STATES

## Abstract

**Introduction:**

Several cytotoxic anticancer drugs inhibit DNA replication and/or mitosis, while EGFR tyrosine kinase inhibitors inactivate EGFR signalling in cancer cell. Both types of anticancer drugs improve the overall survival of the patients with non-small-cell lung cancer (NSCLC), although tumors often become refractory to this treatment. Despite several mechanisms by which the tumors become resistant having been described the effect of these compounds on anti-tumor immunity remains largely unknown.

**Methods:**

This study examines the effect of the cytotoxic drug Gemcitabine and the EGFR tyrosine kinase inhibitor Gefitinib on the expression of NK group 2 member D (NKG2D) ligands as well as the sensitivity of NSCLC cells to the NK-mediated lysis.

**Results:**

We demonstrate that Gemcitabine treatment leads to an enhanced expression, while Gefitinib downregulated the expression of molecules that act as key ligands for the activating receptor NKG2D and promote NK cell-mediated recognition and cytolysis. Gemcitabine activated ATM and ATM- and Rad-3-related protein kinase (ATR) pathways. The Gemcitabine-induced phosphorylation of ATM as well as the upregulation of the NKG2D ligand expression could be blocked by an ATM-ATR inhibitor. In contrast, Gefitinib attenuated NKG2D ligand expression. Silencing EGFR using siRNA or addition of the PI3K inhibitor resulted in downregulation of NKG2D ligands. The observations suggest that the EGFR/PI3K pathway also regulates the expression of NKG2D ligands. Additionally, we showed that both ATM-ATR and EGFR regulate MICA/B via miR20a.

**Conclusion:**

In keeping with the effect on NKG2D expression, Gemcitabine enhanced NK cell-mediated cytotoxicity while Gefitinib attenuated NK cell killing in NSCLC cells.

## Introduction

Lung cancer ranks as the most commonly diagnosed cancer and the leading cause globally of cancer-related mortality [1]. Despite improvement in clinical outcomes patients with disseminated disease have a poor prognosis. Cytotoxic anticancer therapy has improved overall survival of patients with non-resectable non-small cell lung cancer (NSCLC) [2]. Additionally, EGFR tyrosine kinase inhibitors (EGFR-TKI) have increased the median survival time of advanced patients with EGFR-mutated NSCLC to >24 months [3]. However, both types of drugs however can give rise to refractory tumors.

It is established that the development and progression of tumors might be caused by their escape from immune surveillance and destruction by the host immunity. NK cells play an important role in immunosurveillance [4]. The NK cell activity is promoted via the NK group 2, member D (NKG2D) receptors on NK cells and the engagement of NKG2D with its ligands enhances the cell-mediated cytotoxicity and cytokine release [5–7]. In contrast, NK cell activity could be inhibited by the interaction of the killer-cell immunoglobulin-like receptor (KIR) on NK cells with the major histocompatibility complex (MHC) class I molecules thereby preventing cytotoxicity against normal self [8, 9]. An important NKG2D ligand is the MHC class I chain A and B (MICA and MICB) [5] and UL16-binding proteins (ULBPs) [10], which are expressed at low levels on the non-malignant cell types while primary tumor cells and tumor cell lines frequently express NKG2D ligands [11]. The mechanisms that control the expression of NKG2D ligands are poorly understood. Improving the knowledge of NKG2D ligand expression is important, since it is well established that NKG2D ligands play an important role in the recognition of tumor cells by NK cells [12]. In addition, tumor cells expressing NKG2D ligand can become susceptible to NK cell killing even in the presence of normal MHC class I expression [5, 13]. Thus, the balance between NKG2D ligands and MHC class I expression on transformed cells is important for their survival during immune surveillance of their host [14]. Even so tumor cells could evade immune recognition by downregulating NKG2D ligands expression.

The NKG2D ligands have been described as stress-related proteins, which can be induced by activation of the DNA damage pathway induced by ionizing radiation and inhibitors of DNA replication [15]. The underlying molecular mechanisms of modulating NKG2D ligand expression are diverse. A direct control of MICA by the BCR/ABL oncogene in chronic myelogenous leukemia had been described [16], while the HER2-HER3 signalling was involved in the regulation of MICA/B expression in breast cancer cell lines [17, 18]. Recently, there exists evidence that microRNAs (miRs), such as miR20a, miR–93, miR-106b, miR–372 and miR-520d could have been described to control the constitutive and/or IFN-γ-induced expression of NKG2D ligands in tumor and virus-infected cells [19–21], and the knock down of the miR biogenesis enzyme DICER upregulated MICA/B expression [22].

Cytotoxic anticancer drugs inhibit DNA replication [23] or mitosis [24, 25] resulting in apoptosis in several types of cancers including NSCLC [26], while EGFR-TKI inactivates EGFR signalling in NSCLC cells [27]. Since the NK cell-mediated cytotoxicity is regulated by the balance between activating and inhibiting signals,[28] it is of particular interest to understand if cytotoxic anticancer drugs and EGFR-TKI affect the expression of activating NK cell receptors in NSCLC cells. In this report, we demonstrate that Gemcitabine upregulates NKG2D ligand expression on tumor cells via the activation of ataxia-telangiectasia-mutated (ATM) and ATM-and Rad-3-related protein kinases (ATR) pathway resulting in an enhanced NK cell-mediated cytotoxicity, while Gefitinib downregulates NKG2D ligands expression via inhibition of the EGFR/PI3K signalling thereby hindering NK cell killing. These findings emphasize the importance of immune selection and immune escape in addition to drug resistance for the progress of NSCLC.

## Materials and Methods

### Ethics Statement

This study includes blood samples from buffy coats of blood provided by the blood bank at the Karolinska University Hospital. The buffy coats were provided anonymously. Therefore informed consent was not required. The ethical research committee at Karolinska University Hospital approved the study (No. 20010305, 01–50). This study also includes blood samples at Kawasaki Medical School. The blood samples were collected only from researchers who engaged this study; hence written informed consent was not required. The ethical research committee at Kawasaki Medical School approved the study (No. 1217–3). Both ethical research committees approved this consent procedure.

### Cell culture and reagent

The human NSCLC cell lines RERF-LC-AI, RERF-LC-KJ, and LC2/Ad were obtained from Riken BRC through the National Bio-Resource Project of the MEXT (Tsukuba, Japan). The A549 cells were purchased from both Riken BRC and American Type Culture Collection (Manassas, VA), while the PC–9 cell line was obtained from IBL cell bank (Gunma, Japan). The genotypes of all cell lines were identified with STR Identifier (Applied Biosystems) or PowerPlex 16 STR system (Promega). All the cell lines were maintained in culture in RPMI 1640 medium with 2mM L-glutamine (Invitrogen) supplemented with 10% FBS (Sigma-Aldrich) (A549, RERF-LC-AI, RERF-LC-KJ and PC–9) or 15% FBS (LC2/ad) and 50 U/ml penicillin streptomycin (Sigma-Aldrich) at 37°C in a humidified atmosphere with 5% CO_2_. For cell culture work, Gefitinib (Cayman, #13166), Docetaxel (Enzo Life Sciences, BML-T129), LY294002 (Cayman, #70920), PD98059 (Cayman, #10006726) and KU55933 (Tocris Bioscience, #3544) were dissolved in DMSO (Sigma-Aldrich). Gemcitabine (Tocris Bioscience, #3259), Pemetrexed (Santa Cruz, #sc–219564), Vinorelbine (Santa Cruz, #sc–216059), Caffeine (Tocris Bioscience, #2793) and N-acetyl-L-cysteine (NAC) (Sigma-Aldrich, #A9165) were dissolved in PBS (-).

### WST cell proliferation assay

NSCLC cells were cultured in triplicate wells of 96-well flat-bottomed plates (Asahi Glass) with 1-100nM Gemcitabine, Pemetrexed, Docetaxel, or Vinorelbine in 100μl of culture medium for 48 hours, then 10μl of WST reagent (Roche) were added to wells for 4 hours according to the manufacturer’s protocol. Colorimetric reaction was measured by a spectral scanning multimode reader Varioskan Flash (Thermo Scientific). The chemotherapeutic reagent-mediated inhibition of cell proliferation was calculated with following formula: 100 × (absorbance of the wells treated with reagent/ absorbance of wells treated with medium or DMSO as no treatment control).

### Analysis of cell surface molecules by flow cytometry

Tumor cells were stained with fluorochrome-conjugated antibodies as previously described [17]. The following antibodies were used for staining; Alexa 488 or allophycocyanin-labeled HLA-A,B,C (clone G46-2.6; BioLegend), PE- or allophycocyanin-labeled MICA (clone 159227; R&D systems), allophycocyanin-labeled MICB (clone 236511; R&D systems), Alexa 488-labeled ULBP–1 (clone 170818; R&D systems), PE- or allophycocyanin-labeled ULBP–2/5/6 (clone 165903; R&D systems), PE-labeled ULBP–3 (clone 166510; R&D systems), allophycocyanin-labeled EGFR (clone AY13; BioLegend) as well as Alexa 488-, PE- and allophycocyanin-labeled anti-mouse IgG1κ (clone MOPC–21; BioLegend) or IgG2bκ (clone MOPC–173; BioLegend) as isotype controls. Stained cells were acquired on a FACSCalibur cytometer (BD Biosciences) or FACSCanto II (BD Biosciences) and analyzed using Flowjo software 6.4.7 (Treestar). The increase in mean fluorescence intensity (ΔMFI) was calculated as: (MFI with specific mAb–MFI with isotype control)/ MFI with isotype control. Relative MFI (rMFI) values were calculated to compare the differences between ΔMFI of a specific treatment and control as: 100 × (ΔMFI of specific treatment/ ΔMFI of control treatment) [16].

### Analysis of DNA stress by flow cytometry

Tumor cells were washed in PBS (-), detached with EDTA and 0.25% Trypsin (Sigma-Aldrich), and analyzed for DNA stress using a FlowCellect Multi-Color DNA Damage Response Kit (Millipore) according to the manufacturer’s protocol. The expression of phosphorylated ATM was assessed by flow cytometry.

### siRNA assay

Tumor cells were grown in 12-well plates and transfected after reaching 50% confluency with siRNA targeting EGFR (Dharmacon, #L-003114-00-0005) along with the control siRNA (Dharmacon; #D-001810-10-05), or siRNA targeting ATM (Santa Cruz, #sc–29761) along with the control siRNA (Santa Cruz, #sc–37007), respectively. Cells were transfected in Opti-MEM I cell culture medium (Invitrogen) using Lipofectamine 2000 (Invitrogen) according to the manufacturer’s protocol. After 48 hours, transfected cells were used for further experiments.

### miRNA quantitative PCR (qPCR)

Total miRNA and mRNA of non-treated or treated cell lines was isolated using the TRIzol reagent (Invitrogen) according the manufacturer’s protocol. Real-time quantification of miRNAs was assayed as previously described [29]. Briefly, 200 ng of total RNA was reverse transcribed into complementary DNA and amplified using specific primers summarized in (S1 Table). Quantification of real-time PCR was performed and evaluated on a BioRad iCycler system (BioRad) using the Platinum SYBR Green qPCR SuperMix-UDG (Invitrogen) and a primer mixture of 2:1 (fw:rev). The absolute copy number was calculated using a standard miRNA with known copy numbers. All qPCR analyses were performed in duplicate using RNA from three independent experiments.

### miR inhibition/mimic assay

Tumor cells were transfected in Opti-MEM I cell culture medium (Invotrogen) using Lipofectamine 2000 transfection protocol (Invitrogen) with 5’-fluorescein amidite (FAM) labeled miR20a mimic (miRCURY LNA miR mimic: Has-miR20a-3p, Exiqon, #479995–011) along with 5’-FAM labeled miR inhibitor control (miRCURY LNA miR mimic: Cel-miR39-3p, Exiqon, #479995–011) or 5’-FAM labeled miR20a inhibitor (miRCURY LNA inhibitor: has-miR20a-5p, Exiqon, #4100414–011) along with 5’-FAM labeled miR inhibitor control (miRCURY LNA inhibitor: Negative control A, Exiqon, #199006–011), respectively. After 24 hours or 48 hours for assays with miR inhibitor or ones with miR mimic, respectively, transfected cells were treated with 10 nM of Gemcitabine or 1 μM of Gefitinib. Again after 24 hours, cells were analyzed for cell surface molecule expression using flow cytometry as described above. The efficacy of transfection was confirmed using Flow cytometry. The sequence of miR mimics and inhibitors are summarized in (S2 Table).

### Western blot analysis

Cells were lysed in CelLytic (Sigma-Aldrich) buffer with protease inhibitor (Protease Inhibitor Cocktail; Sigma-Aldrich) and phosphatase inhibitor (Phosphatase Inhibitor Cocktail 2; Sigma-Aldrich) then the protein concentrations were analyzed using a BCA protein assay (Thermo Scientific or Takara Bio) according to the manufacturer’s protocol. Western blotting to assess the expression of EGFR, phosphorylated EGFR (pEGFR) and β-actin was performed as previously described [17]. Briefly, equal amounts of protein were separated on 4% to 12% NuPage Bis-Tris acrylamide gels (Invitrogen) and transferred to polyvinylidene difluoride (PVDF) membranes (Immobilon-P; Millipore). On the other hand, to test ATM expression, equal amounts of protein were separated on 3% to 8% NuPage Tris-Acetate gels and transferred to PVDF membranes (Life Technologies) using iBlot2 dry blotting system (Life Technologies) according the manufacturer’s protocol. Blots were blocked in PBS (-) with 0.05% of Tween 20 (Sigma-Aldrich) buffer (PBS-T) with 2% non-fat dry milk, followed by incubation over night at 4°C in PBS-T with 2% non-fat dry milk and primary antibody against: EGFR, pEGFR (Tyr1068) (Cell Signaling Technology), ATM (Santa Cruz) and β-actin (Sigma-Aldrich). After two washes in PBS-T, membranes were incubated with HRP-linked goat anti-rabbit or anti-mouse IgG antibodies (Cell Signaling Technology) for 1 hour at room temperature. Blots were visualized by enhanced chemiluminescence with an ECL plus or ECL prime system (GE Healthcare), and images were captured using a LAS–1000 or LAS–4000 camera system (Fujifilm). Membranes were stripped in PBS (-) with 2% SDS (Invitrogen) and 0.7% 2-mercaptoethanol (Sigma-Aldrich) for 30 min at 50°C, and reprobed up to three times.

### NK cell-mediated cytotoxicity assay and NK cell-degranulation assay

Peripheral blood mononuclear cells (PBMCs) were collected from healthy donor through density centrifugation. Negatively purified NK cells (NK cell isolation kit; Stem Cell Technology) were incubated overnight with 100 IU/ml of human recombinant IL–2 (Teceleukin, Shionogi). The phenotype of the cells (CD3-CD56+) were confirmed by flow cytometric analysis using Alexa 488-labeled anti-CD3 (clone HIT3a; BioLegends) and allophycocyanin-labeled anti-CD56 (clone HCD56; BioLegends) antibodies. Both untreated and treated target cells were tested for sensitivity to NK cell-mediated lysis using a LDH release assay (CytoTox 96 Non-radioactive Cytotoxicity assay, Promega) according to the manufacturer’s protocol. LDH release in the supernatants was measured by a spectral scanning multimode reader Varioskan Flash (Thermo Scientific). The percentage of specific lysis was calculated as follows: 100 × (experimental release–spontaneous release) / (maximum release–spontaneous release). For NK cell degranulation assay, NK cells were co-incubated with target cells at ratio 2:1 in a final volume of 200 μl in round-bottomed 96-well plates at 37°C and 5% CO2 for 4h. PE-conjugated anti-CD107a mAb (clone H4A3; BioLegends) or the corresponding isotype control was added at the initiation of the assay. After 1h of co-incubation, Monensin solution (BioLegend) was added at 1:1000 dilution. Surface staining was done by incubating the cells with anti-CD56 mAb for 15 min at 4°C. The cells were washed, resuspended in PBS (–) with 1% FCS and 2% paraformaldehyde, and then analyzed by flow cytometry. To analyze the involvement of NKG2D in the cytotoxicity and degranulation activity of NK cells, effector cells were co-incubated with 20 μg/ml of anti-NKG2D blocking antibody (clone 1D11; BioLegend) or an isotype-matched control antibody (clone 11711; R&D systems).

### Statistics

Differences in means were evaluated with Student *t*-test. All analyses were performed at a significance level of 5% (p< 0.05) using GraphPad Prism 5 (GraphPad Software Inc.).

## Results

### Constitutive expression of MHC class I molecules MICA, MICB, ULBP–1, ULBP–2/5/6 and ULBP–3 in NSCLC cell lines

**Fig 1:**
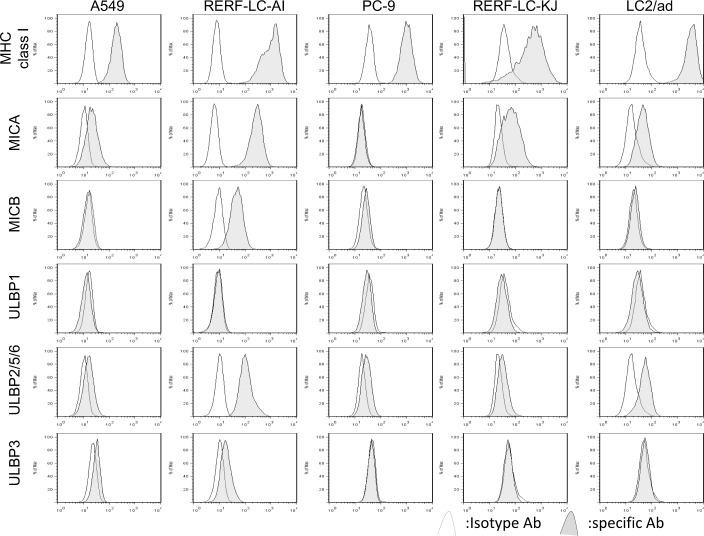
The expressions of MHC class I, MICA, MICB, ULBP1, ULBP2/5/6, and ULBP3 in non-small-cell lung cancer cell lines. The basal expression of each cell surface molecules was assessed by flow cytometric analysis. The cells were stained with isotype control (white histograms) or specific antibody for the indicated molecule (gray histograms).

The basal expressions levels of MHC class I molecules and the different NKG2D ligands MICA, MICB, ULBP–1, ULBP–2/5/6 and ULBP–3 were determined in 5 NSCLC cell lines using flow cytometry. As shown in Fig 1, ULBP–2/5/6 was commonly expressed in all NSCLC lines analyzed. Both MICA and ULBP–1 were expressed in 4 of 5 cell lines, but the expression level of ULBP–1 was low. In contrast, both MICB and ULBP–3 were expressed in only 2 of 5 cell lines, but MICB is highly expressed in RERF-LC-AI cells. Based on the expression pattern we focused on the expressions of MICA, MICB and ULBP–2/5/6 for subsequent experiments.

### Cytotoxic anticancer drugs enhanced MICA/B and ULBPs expressions in NSCLC cells

**Fig 2:**
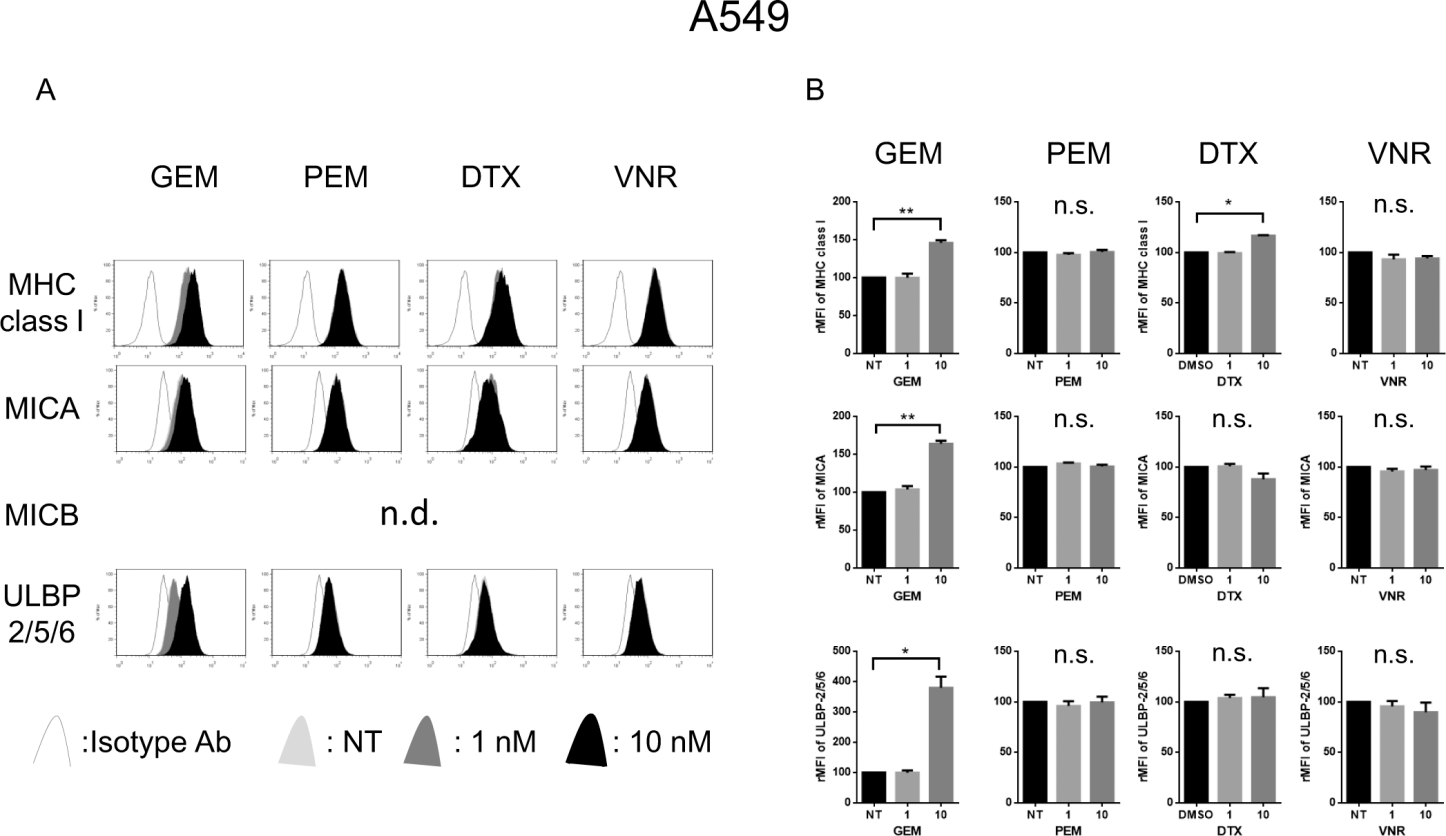
The expression of NKG2D ligands are upregulated by Gemcitabine in A549 cells. (A): A549 cells were treated with or without 1 to 10nM of Gemcitabine (GEM), Pemetrexed (PEM), Docetaxel (DTX) or Vinorelbine (VNR) for 24 hours, then the expression of MHC class I molecules and NKG2D ligands were assessed by flow cytometry. (B): The relative MFI (rMFI) of MHC class I molecules and NKG2D ligands were calculated based on at least three independent experiments and evaluated with a Student *t*-test. Bars, SEM. * -p<0.05 and ** -p<0.01.

We next examined the effect of the cytotoxic reagents on the proliferation of NSCLC cells using the WST assay demonstrating that A549 cells were resistant to several cytotoxic reagents compared with other cell lines (S1 Fig). To analyze the ability of cytotoxic anticancer drugs to influence the MHC class I antigen and NKG2D ligand expression, the 5 NSCLC cell lines were left untreated (NT) or treated with 1 or 10 nM of Gemcitabine, Pemetrexed, Docetaxel, or Vinorelbine, respectively, for 24 hours before the expression of each molecule was assessed. Gemcitabine upregulated the expression of MHC class I molecules, MICA, and ULBP–2/5/6 in A549 cells (Fig 2A and 2B), and the expression of MICA, MICB, and ULBP–2/5/6 in RERF-LC-AI cells (S2A Fig). Docetaxel upregulated MHC class I molecules in A549 cells (Fig 2A and 2B), ULBP–2/5/6 in RERF-LC-AI (S2A Fig), and both MICB and ULBP–2/5/6 in PC–9 cells (S2B Fig). Neither Pemetrexed nor Vinorelbine could enhance the expression of both MHC class I molecules and NKG2D ligands in these 3 cell lines (Fig 2A and 2B, S2A and S2B Fig) and we could not see any effect on the expression of MHC class I molecules and NKG2D ligands in neither RERF-LC-KJ nor LC2/ad cells treated with any of the cytotoxic drugs (S2C and S2D Fig).

### Gemcitabine-induced NKG2D ligand expression is regulated by DNA stress-induced ATM-ATR signalling

**Fig 3:**
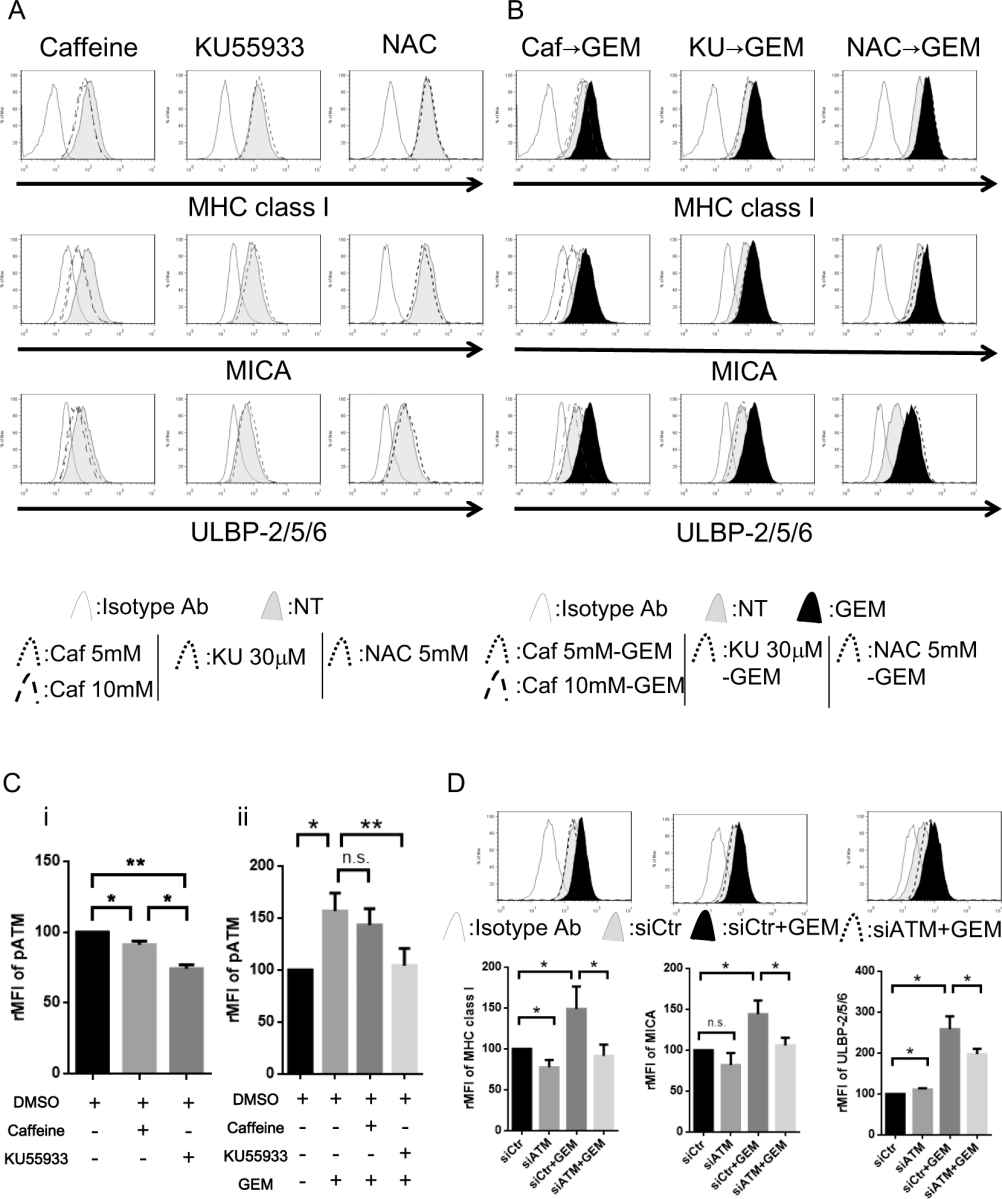
Gemcitabine-induced upregulation of the expression of NKG2D ligands is regulated via ATM-ATR pathway in A549 cells. (A): Histograms demonstrating MHC class I molecules and NKG2D ligands in A549 cells treated with 5, 10 mM of Caffeine or 30μM of KU55933 or 5mM of N-acetyl-L-cysteine (NAC) for 24 hours. (B): Histograms demonstrating MHC class I and NKG2D ligands in A549 cells pretreated with Caffeine or KU55933 or NAC for 2 hours followed by Gemcitabine (GEM) at 10 nM for 24 hours. Data are representative of three independent experiments. NT: no treatment control. (Ci) A549 cells were treated with Caffeine or KU55933 or for 24 hours. The phosphorylated-ATM (pATM) was assessed by flow cytometry then the effects on the expressions of pATM treated with Caffeine or KU55933 were shown as the relative MFI (rMFI) mean values of four independent experiments and evaluated with Student *t*-test. (ii) A549 cells were pretreated with Caffeine or KU55933 for 2 hours followed by Gemcitabine for 24 hours then the pATM was assessed by flow cytometry. The effects on the expressions of pATM were shown as the rMFI mean values of at least three independent experiments and evaluated with Student *t*-test. (D): MHC class I molecules and NKG2D ligands in A549 cells treated with siRNA of ATM for 24 hours followed by Gemcitabine (GEM) at 10 nM for 24 hours. Representative histograms of three independent experiments are shown. The relative MFI (rMFI) of MHC class I molecules and NKG2D ligands were calculated based on at least three independent experiments and evaluated with a Student *t*-test. Bars, SEM. * -p<0.05 and ** -p<0.01.

It has been demonstrated that NKG2D ligand expression can be induced by activation of the DNA stress sensing ATM-ATR [15] or the NKG2D ligand gene transcription via oxidative stress [30]. To determine whether ATM-ATR pathways regulate the NKG2D ligand expression in NSCLC, A549 cells were treated with the ATM-ATR inhibitors Caffeine [31, 32] or KU55933 [33]. In concordance with recently published reports [15, 17], the baseline expression of NKG2D ligands was downregulated by Caffeine, but was not downregulated by KU55933 or by the anti-oxidative reagent NAC in A549 cells (Fig 3A). The upregulation of NKG2D ligands which was observed in A549 cells following 24 hour exposure to 10 nM of Gemcitabine (Fig 2A and 2B) was blocked both by Caffeine and KU55933 treatment but marginal effect by NAC (Fig 3B), suggested that Gemcitabine-induced NKG2D ligand was mainly regulated by ATM-ATR pathway but not by oxidative stress. In subsequent experiments, we explored whether the ATM-ATR signalling would affect NKG2D ligand expression to delineate why Caffeine blocked both basal and Gemcitabine-induced NKG2D ligands while KU55933 blocked only Gemcitabine-induced NKG2D ligands. Flow cytometric analysis of phosphorylated ATM showed that the ATM-ATR inhibitors KU55933 clearly blocked phosphorylation of ATM, while Caffeine had a smaller effect on basal (Fig 3Ci) and Gemcitabine-induced (Fig 3Cii) phosphorylation of ATM than KU55933. To investigate this further, the expression of NKG2D ligands were analyzed in A549 cells treated with ATM-specific siRNA. As shown by Western blot analysis, the ATM expression in A549 cells was diminished by ATM siRNA (S3 Fig). In line with the experiments using the ATM inhibitor KU55933, ATM-specific siRNA did not decrease basal expression of NKG2D ligands, but clearly blocked Gemcitabine-induced NKG2D ligands in A549 cells (Fig 3D).

It has been reported that Caffeine inhibits ATM-ATR pathway [31] as well as PI3K pathway [34], which are also known as regulators of NKG2D ligands [16, 17]. Our findings suggested that the Gemcitabine-induced NKG2D ligands were mainly regulated via ATM-ATR pathway, but basal expression of NKG2D ligand is regulated via other mechanisms.

### Gefitinib downregulated NKG2D ligands expression via blocking the downstream signalling of EGFR, mainly PI3K-AKT pathway

**Fig 4:**
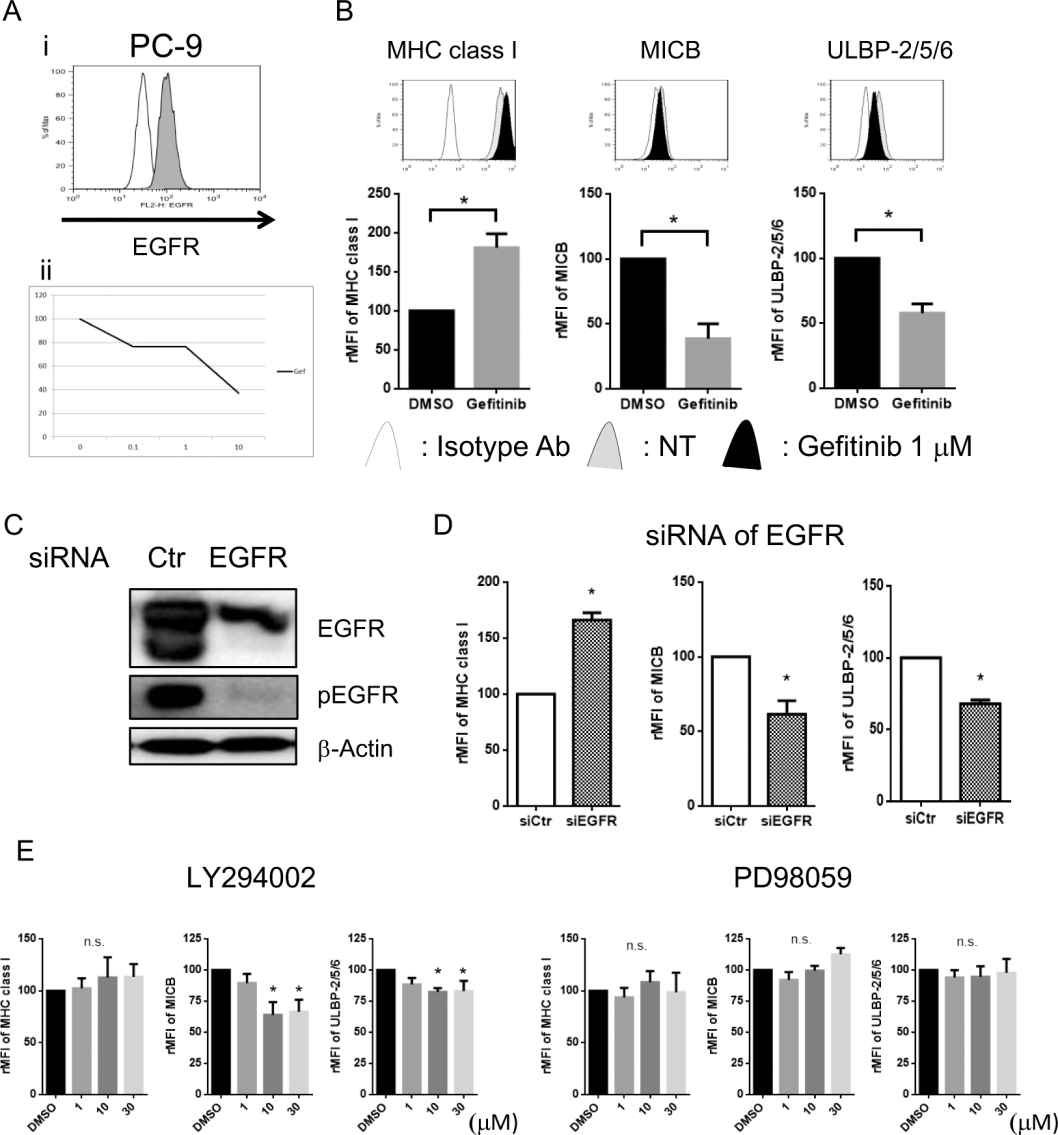
The expression of NKG2D ligands are regulated by EGFR/PI3K/AKT pathway in PC–9 cells. (Ai): The basal expression of EGFR was assessed by flow cytometry in PC–9 cells. (ii): WST cell proliferation assay showed PC–9 cells were sensitive to Gefitinib (Gef). (B): PC–9 cells were treated with or without 1 μM of Gef for 24 hours. MHC class I molecules and NKG2D ligands were assessed by flow cytometry. The representative histograms from three independent experiments were shown. The relative MFI (rMFI) of MHC class I molecules, MICB, and ULBP–2/5/6 were calculated based on at least three independent experiments and evaluated with a Student *t*-test. **(**C): PC–9 cells were transfected with siRNA targeting EGFR (siEGFR) or control siRNA (siCtr) as control for 48 hours. The expressions of EGFR, phosphorylated EGFR (pEGFR) and β-actin were assessed by Western blot analyses. Data are presented as representatives of three independent experiments. (D): The expressions of MHC class I, MICB and ULBP2/5/6 were assessed by flow cytometry, then the effects on the expressions of these molecules treated with siRNA of EGFR were shown as the rMFI mean values of three independent experiments and evaluated with Student *t*-test. (E): PC–9 cells were cultured with various concentration of the PI3K inhibitor LY294002, MEK1 inhibitor PD98059 or DMSO (0.01%) as control. The effects on the expressions of MICB and ULBP2/5/6 treated with each inhibitor are shown as the rMFI mean values of three independent experiments and evaluated with Student *t*-test. Bars, SEM. * -p<0.05

Next, the ability of the EGFR-TKI Gefitinib was examined for its ability to influence MHC class I antigen and NKG2D ligand expression in these cell lines. Although it was previously reported that 10 μM of Gefitinib upregulated NKG2D ligands in lung cancer cell lines [35], pharmacokinetic analysis showed the serum concentration of Gefitinib reached less than 1 μM in the clinical setting [36]. Therefore we assessed the influence of Gefitinib at 1 μM on NKG2D ligand expression. Both PC–9 (Fig 4Ai) and A549 cells (S4A Fig) expressed EGFR, but PC–9 is sensitive (Fig 4Aii), while A549 is resistant to Gefitinib (S4A Fig). In line with a previous report [37], Gefitinib upregulated the expression of MHC class I antigens in both PC–9 and A549 cells. Surprisingly, Gefitinib downregulated the basal expressions of MICB and ULBP–2/5/6 in PC–9 cells (Fig 4B) and MICA in A549 (S4B Fig), although Gefitinib did not show any effect in the other carcinoma lines tested (S5 Fig).

To further assess and confirm the influence of EGFR signalling on the expression of NKG2D ligands, EGFR expression was silenced in both PC–9 and A549 cells using RNAi. The expressions of EGFR in both cell lines were diminished by EGFR siRNA as measured by Western blot, which was accompanied by the inhibition of the phosphorylation of EGFR in PC–9 cells (Fig 4C) and A549 cells (S4C Fig). In line with previous reports [37], EGFR siRNA upregulated the expression of MHC class I molecule, while a significant downregulation of the expression of MICB and ULBP–2/5/6 was observed in PC–9 cells (Fig 4D) and the downregulation of MICA in A549 (S4D Fig). These findings suggested that the EGFR directly regulated the expression of NKG2D ligands. It has been previously demonstrated that the oncogenes BCL/ABL in chronic myelogenous leukemia [16] and HER3 in breast cancer [17, 18] regulated NKG2D ligands expression via PI3K/AKT signalling. EGFR-TKI therapies improve the outcome of NSCLC patients while EGFR driver mutations help cell survival and proliferation [3]. Since the PI3K/AKT and the RAS/MAPK pathways are the major downstream pathways of EGFR signaling [38], we evaluated whether these pathways regulate the expression of NKG2D ligands in the NSCLC cells. PC–9 cells were treated with the PI3K inhibitor LY294002 [39] or the MEK inhibitor PD98059 [40]. In line with our earlier findings with the breast carcinoma cell lines [17], LY294002 strongly downregulated NKG2D ligands, but PD98059 showed smaller effect than LY294002 on the expression of NKG2D ligands in both A549 (Fig 4E) and PC–9 cells (S4E Fig) suggesting that the EGFR signalling maintained the basal expression of NKG2D ligands via mainly PI3K-AKT pathway in both 2 cell lines.

Gemcitabine enhanced the expression of NKG2D ligands, while Gefitinib attenuated them in A549 cells. Since combination therapy regimens are useful in clinic, A549 cells were treated with both Gemcitabine and Gefitinib for 24 hours. As expected the combination treatment of Gemcitabine and Gefitinib enhanced the expression of MHC class I antigens, while the Gemcitabine induced MICA/B reverted by co-treatment with Gefitinib in A549 cells (S6 Fig).

### Both ATM-ATR and EGFR pathway regulated MICA/B expression via miR20a

**Fig 5:**
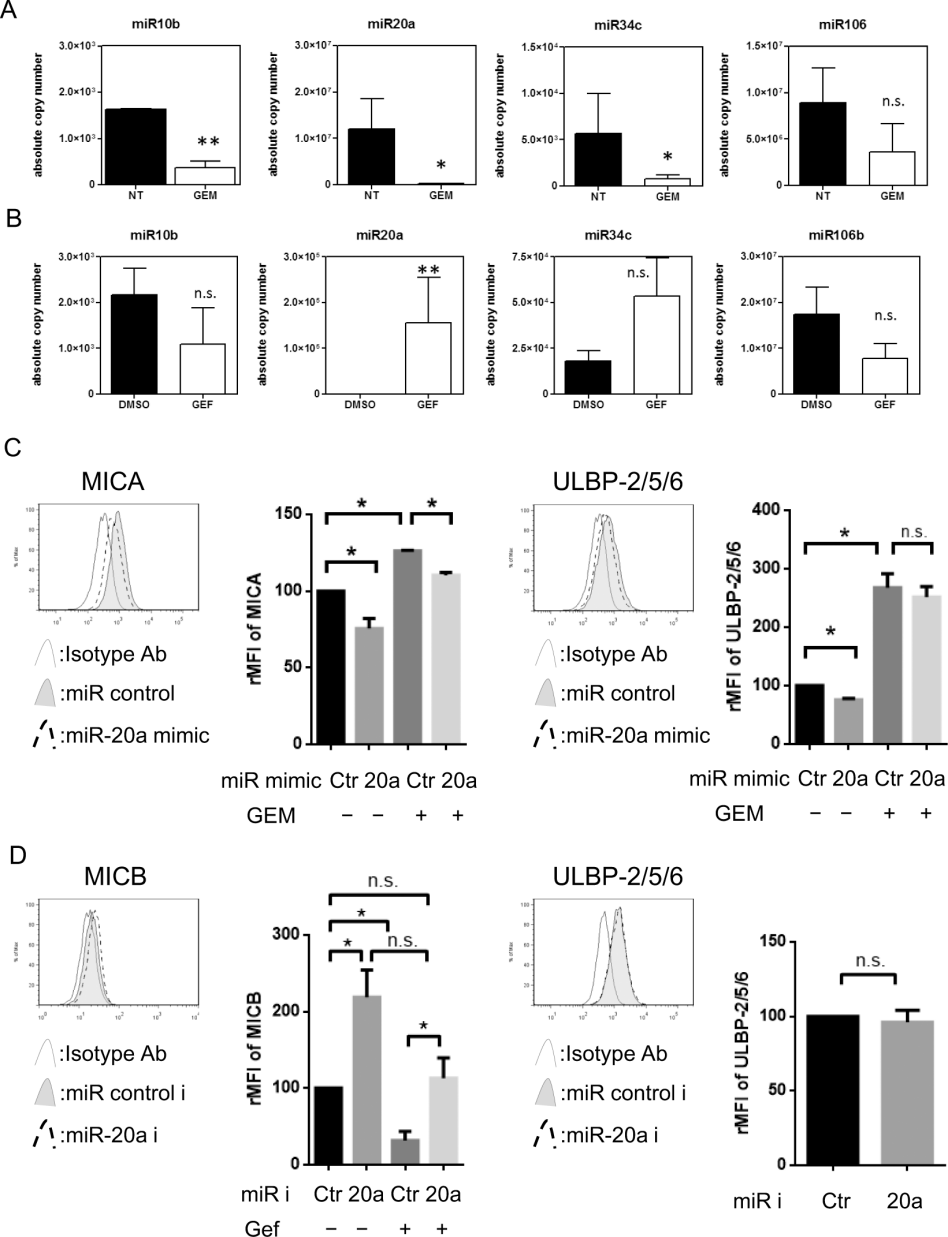
The expressions of miRs in NSCLC cells treated with Gemcitabine or Gefitinib. (A): A549 cells were treated with Gemcitabine for 24h then miR10b, miR20a, miR34c and miR106 expression was evaluated using quantitative real-time PCR as described in Materials and Methods. The results represent the mean of duplicates of 3 independent experiments. (B): PC–9 cells were treated with Gefitinib for 24h then miR10b, miR20a, miR34c and miR106 were evaluated using quantitative real-time PCR for 3 independent experiments. Differences in means were evaluated with Student *t*-test. (C): NKG2D ligands MICA and ULBP–2/5/6 were assessed by flow cytometry in A549 cells treated with miR20a mimic for 48 hours followed by Gemcitabine (GEM) at 10 nM for 24 hours. Representative histograms of three independent experiments and the relative MFI (rMFI) of NKG2D ligands were calculated based on three independent experiments and evaluated with a Student *t*-test. (D): NKG2D ligands MICB and ULBP–2/5/6 were assessed by flow cytometry in PC–9 cells treated with miR20a inhibitor for 24 hours followed by Gefitinib (Gef) at 1 μM for 24 hours. Representative histograms of three independent experiments and the relative MFI (rMFI) of NKG2D ligands were calculated based on three independent experiments and evaluated with a Student *t*-test. Bars, SEM. * -p<0.05 and ** -p<0.01.

Recently miRNA has been demonstrated to downregulate the expression of several molecules including NKG2D ligands [20]. In order to determine whether ATM-ATR or EGFR signalling regulates NKG2D ligand expression by miRNA, the copy number of miRNA was assessed in NSCLC cells treated with Gemcitabine or Gefitinib. Interestingly, miR10b and miR20a expression decreased in both A549 (Fig 5A) and RERF-LC-AI cells (S7A Fig) treated with Gemcitabine, suggesting that Gemcitabine-induced ATM-ATR signalling regulates NKG2D ligand expression via miR10b and miR20a. On the other hand, Gefitinib upregulated miR20a in PC–9 cells (Fig 5B) although only a marginal effect was seen for miR20a in A549 cells (S7B Fig). To assess the function of miR20a on the expression of NKG2D ligands, A549 cells were transfected with control or miR20a mimic then treated with Gemcitabine. The transfection efficacy of control miR or miR20a mimic were evaluated by flow cytometry (S8A Fig). The miR20a mimic downregulated MICA expression and blocked Gemcitabine-induced MICA, while miR20a mimic has no effect on Gemcitabine-induced ULBP–2/5/6 in A549 cells (Fig 5C). On the other hand, PC–9 cells were pretreated with miR20a inhibitor then exposed with Gefitinib. Transfection of miR20a inhibitor was confirmed by flow cytometry (S8B Fig) and the miR20a inhibitor clearly upregulated the expression of MICB and blocked Gefitinib-induced inhibition of MICB expression, while miR20a inhibitor did not affect ULBP–2/5/6 expression in PC–9 cells (Fig 5D). These results suggest that both ATM-ATR and EGFR pathway regulated MICA/B expression via miR20a, while ULBP–2/5/6 expression might be regulated by other miRs.

### Gemcitabine-induced NK cell-mediated cytotoxicity is NKG2D-dependent while Gefitinib attenuated NK killing

**Fig 6:**
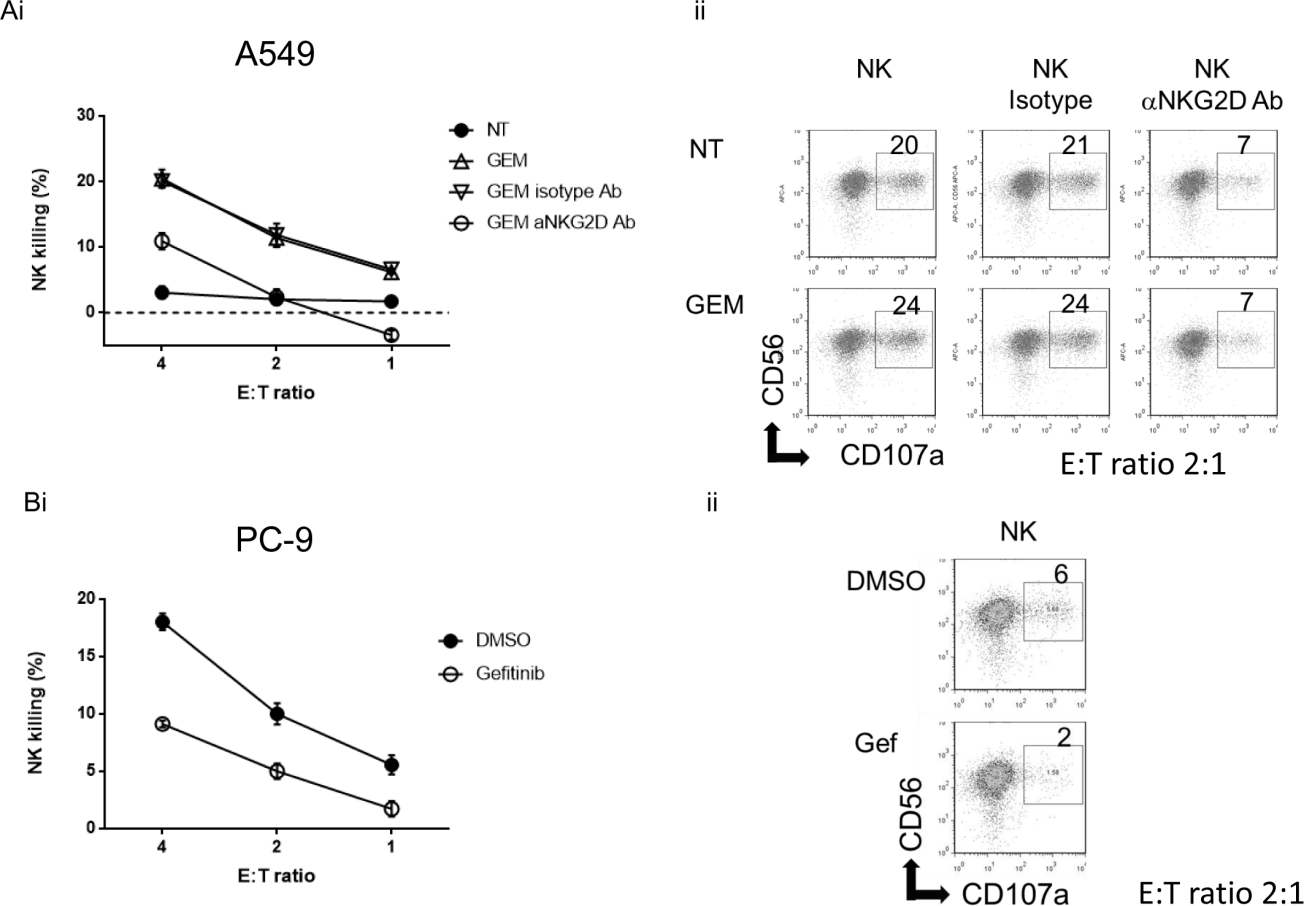
Effects of Gemcitabine or Gefitinib on NK cell-mediated cytotoxicity and NK cell degranulation. (Ai): A549 cells with or without Gemcitabine (GEM) (10 nM) for 24 hours were subjected to LDH release assay for 4 hours using IL–2 activated NK cells as effector cells. The IL–2 activated NK cells were pretreated with blocking antibody of NKG2D or isotype control for 30 min before the cytotoxicity assay. Data are presented as mean of triplicate samples and are representative of three independent experiments. (ii): IL–2 activated NK cells were pretreated with blocking antibody of NKG2D or isotype control before NK cell degranulation assay, then coincubated with A549 cells treated with or without GEM for 4 hours, and NK cell degranulation was evaluated. (Bi): PC–9 cells with or without Gefitinib (Gef) (1 μM) were subjected to LDH release assay for 4 hours using IL–2 activated NK cells as effector cells. Data are presented as mean of triplicate samples and are representative of three independent experiments. (ii): IL–2 activated NK cells were coincubated for 4 hours with PC–9 cells pretreated with or without Gefitinib, and NK cell-degranulation was evaluated. Data are representative of three independent experiments. E:T ratio; effector/target ratio.

NKG2D ligands are engaged by the NKG2D receptor expressed on NK cells and CD8+ T cells [5, 11]. Since Gemcitabine enhanced the expression of NKG2D ligands in NSCLC cells, we evaluated if Gemcitabine would affect their sensitivity to NK cell-mediated cytotoxicity. Tumor cells were treated with 10 nM of Gemcitabine for 24 hours and thereafter their sensitivities to NK cell cytotoxicity and degranulation activity were evaluated using IL–2 activated NK cells. As expected, Gemcitabine treatment enhanced NK cell-mediated killing in A549 cells. To verify that this receptor is involved in the Gemcitabine-induced sensitivity to NK cell killing, purified NK cells were pretreated with anti-NKG2D blocking antibody before the LDH release assay or NK cell degranulation assay. The anti-NKG2D blocking antibody inhibited Gemcitabine-induced NK cell-mediated lysis as well as CD107a expression in NK cells against the A549 tumor target, while treatment with an isotype control antibody had no effect (Fig 6Ai and 6Aii), indicating that the Gemcitabine-induced NK cell-mediated cytotoxicity was dependent on NKG2D-NKG2D ligand interaction. In spite of the enhancement of MHC class I molecules in A549 cells treatment with Gemcitabine, this treatment enhanced NK killing on tested target cells. This argues for a direct causal relationship between the up-regulation of NKG2D ligand expression by Gemcitabine and the increase in tumor NK sensitivity.

**Fig 7:**
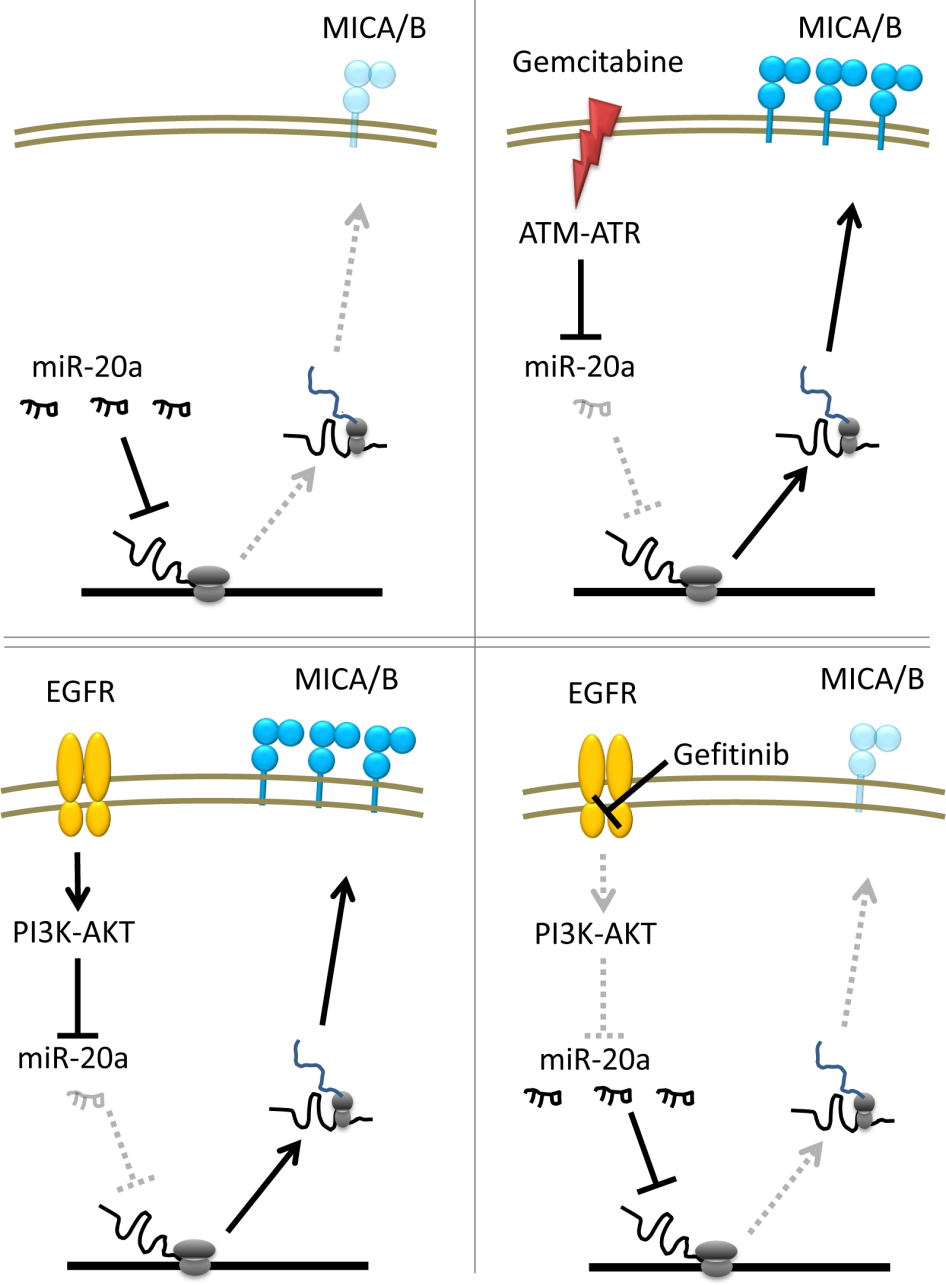
Schematic diagram summarized how ATM-ATR and EGFR pathway regulate MICA/B expression in NSCLC cells.

Next, we evaluated if Gefitinib would affect their sensitivity to NK cell-mediated cytotoxicity, since Gefitinib downregulated the expression of NKG2D ligands in PC–9 cells. PC–9 cells were treated with 1 μM of Gefitinib for 24 hours and thereafter their sensitivities to NK cell cytotoxicity and degranulation activity were evaluated using NK cells. As expected, Gefitinib attenuated NK cell-mediated killing and NK cell degranulation activity in PC–9 cells (Fig 6Bi and 6Bii). Hence, the regulation mechanisms of the expression of NKG2D ligands, which influence NK cell-mediated cytotoxicity, are schematically summarized in Fig 7.

## Discussion

In the present study, we report several findings supporting the induction mechanisms of the NKG2D ligand as well as important roles for regulating NKG2D ligand expression in NSCLC cell lines. Our observations suggest that cytotoxic anticancer drugs can induce the expression of NKG2D ligands. This is in line with the report by Morisaki and coworkers, who reported that Gemcitabine enhances the expression of MICA/B in the hepatocellular carcinoma line HepG2, but the molecular mechanisms of the Gemcitabine-induced MICA/B expression was not analyzed [41]. The regulation of NKG2D ligand expression by Gemcitabine or other cytotoxic drugs has not previously been investigated in NSCLC cells. Therefore this study evaluated whether Gemcitabine enhances NKG2D ligand regulation and determines their impact on NK cell-mediated killing of NSCLC cell lines.

It is reported that the expression of NKG2D ligands is regulated by DNA stress-induced ATM-ATR signaling [15], while other groups including us reported that oncogenes BCL/ABL and HER3 also regulated the expression of NKG2D ligands in transformed cells via mainly PI3K-AKT pathway [16, 17]. The ATM-ATR pathway, which plays a key role in the DNA damage response pathway, is frequently activated in tumor cells and was found to regulate NKG2D ligand via transcriptional regulation [15]. We demonstrated that Gemcitabine activated ATM-ATR signalling, and specific ATM-ATR inhibitor KU55933 blocked Gemcitabine-induced activation of this pathway in A549, in line with a role also for this pathway in maintaining Gemcitabine-induced NKG2D ligands expression. We also assessed that silencing ATM gene could block Gemcitabine-induced NKG2D ligand expression. These findings indicated that Gemcitabine enhanced NKG2D ligands through genotoxic stress induced ATM-ATR signaling, while basal expression of NKG2D ligands might be maintained via other mechanisms including EGFR/ PI3K/AKT in NSCLC cells.

Interestingly, cytotoxic anticancer drugs enhanced several NKG2D ligands while EGFR-TKI Gefitinib down-regulated the expression of NKG2D ligands in NSCLC cell lines. Therefore, a key issue is to determine whether EGFR signaling regulates the expression of NKG2D ligands. Our findings suggested that NKG2D ligand expression is regulated by not only DNA stress-induced ATM-ATR pathway but other signalling such as EGFR in NSCLC cells.

It has been previously reported that several miRNA suppress the expression of NKG2D ligands [20, 42]. We assessed the miRNA expression in NSCLC cells treated with Gemcitabine or Gefitinib. Both substances exert a dissimilar activity on miRNA expression, Gemcitabine, but not Gefitinib decreased both miR10b and miR20a, which were reported as suppressors for expressing NKG2D ligands [43, 44] suggesting that DNA stress may induce NKG2D ligand expression via downregulation of miR10b and miR20a. On the other hand, Gefitinib clearly increased miR20a in PC–9 and marginally increased in A549 cells. Thus, miR20a might be a common regulator for regulating NKG2D ligands. Our functional assay targeting miR20a showed both ATM-ATR and EGFR pathway regulated MICA/B expression via miR20a, although miR20a did not affect ULBP–2/5/6 expression suggesting that ULBP–2/5/6 might be regulated via other miRs.

A critical question was determining the functional consequences of Gemcitabine-induced NKG2D ligand expression on tumor target cells. As anticipated, activation of ATM-ATR signalling by Gemcitabine enhanced sensitivity to NK cell-mediated cytotoxicity in A549 cells. This finding implicated a role for the NKG2D-NKG2D ligand interaction in the modulation of the NK cell sensitivity to A549 cells. This premise was further bolstered by the observation that Gemcitabine-induced increase in NK cell-mediated cytotoxicity could be blocked with an anti-NKG2D blocking antibody.

NKG2D ligands induced by chemotherapeutic reagents such as Gemcitabine could represent one important mechanism, which the tumor cells are eradicated by the innate immune system [12]. Our findings support the conclusion that Gemcitabine enhances NSCLC cells sensitivity for NK cell-mediated cytotoxicity. Conversely, the implication from our results is that EGFR-TKI may suppress tumor sensitivity to NK cells via down-regulation of the expression of NKG2D ligands, as was shown by the attenuation of NK cell-mediated cytotoxicity by Gefitinib.

In theory, our *in vitro* findings suggest that combination regimens with drugs that increase immune-mediated killing together with potent tumoricidal chemotherapy agents may be the optimum approach treating NSCLC. It has been reported that NK cell activity is quite low in patients with poor performance status or advanced disease [45]. The cytotoxic drug-induced NKG2D ligands may help the clearance of tumor cells, but is potentially limited to the patients without NK cell dysfunction. In contrast, Gefitinib attenuates the sensitivity to NK cells, suggesting that therapy targeting EGFR-TKI may be a “double-edged sword” as they inhibit cell proliferation while abetting immune escape from host immunity in NSCLC cells. Regardless, enhancement of NK cell function by NK cell transfer or cytokine administration may be promising strategies to enhance NK killing via cytotoxic drug-induced NKG2D ligands or to overcome the limitation of EGFR-TKI targeting therapy.

## Supporting Information

S1 Fig Chemotheraputic regent inhibited cell proliferation in non-small-cell lung cancer cell lines. Five non-small cell lung cancer cell lines were treated with indicated concentrations of each chemotherapeutic regent for 48h. After the incubation, WST cell proliferation assay were performed. Representative data of three independent experiments are shown.
(TIF)

S2 Fig The expression of MHC class I molecules and NKG2D ligands in RERF-LC-AI, PC–9, RERF-LC-KJ and LC2/ad cells treated with several cytotoxic drugs. RERF-LC-AI **(Figure A)**, PC–9 **(Figure B)**, RERF-LC-KJ **(Figure C)** and LC2/ad **(Figure D)** cells were treated with or without 1 to 10nM of Gemcitabine (GEM), Pemetrexed (PEM), Docetaxel (DTX) or Vinorelbine (VNR) for 24 hours, then the expression of MHC class I molecules and NKG2D ligands were assessed by flow cytometry as shown in the representative histograms from three independent experiments.
(TIF)

S3 Fig The expression of ATM in A549 cells trasnfected with siRNA of ATM. PC–9 cells were transfected with siRNA targeting ATM or control siRNA (siCtr) for 48 hours. The expression levels of ATM and β-actin were assessed by Western blot analyses. Data are presented as representatives of three independent experiments.
(TIF)

S4 Fig The expression of NKG2D ligands are regulated by EGFR/PI3K/AKT pathway in A549 cells. The basal expression of EGFR was assessed by flow cytometry in A549 cells **(Figure Ai)**. WST cell proliferation assay showed A549 cells were resisitant to Gefitinib (Gef) **(Figure Aii)**. A549 cells were treated with or without 1 μM of Gefitinib (Gef) for 24 hours. MHC class I molecules and NKG2D ligands were assessed by flow cytometry. The representative histograms from three independent experiments were shown. The relative MFI (rMFI) of MHC class I molecules, MICA, and ULBP–2/5/6 were calculated based on at least three independent experiments and evaluated with a Student *t*-test **(Figure B)**. A549 cells were transfected with siRNA targeting EGFR (siEGFR) or control siRNA (siCtr) as control for 48 hours. The expressions of EGFR, phosphorylated EGFR (pEGFR) and β-actin were assessed by Western blot analyses. Data are presented as representatives of three independent experiments **(Figure C)**. The expressions of MHC class I and MICA were assessed by flow cytometry, then the effects on the expressions of these molecules treated with siRNA of EGFR were shown as the relative MFI (rMFI) mean values of three independent experiments and evaluated with Student *t*-test. E: A549 cells were cultured with various concentration of the PI3K inhibitor LY294002, MEK1 inhibitor PD98059 or DMSO (0.01%) as control. The effects on the expressions of MHC class I and MICA treated with each inhibitor are shown as the relative MFI (rMFI) mean values of three independent experiments and evaluated with Student *t*-test **(Figure D)**. Bars, SEM. * -p<0.05 and ** -p<0.01.
(TIF)

S5 Fig The expression of MHC class I molecules and NKG2D ligands in RERF-LC-AI, RERF-LC-KJ and LC2/ad cells treated with Gefitinib. RERF-LC-AI, RERF-LC-KJ and LC2/ad cells were treated with or without 1μM of Gefitinib (Gef) for 24 hours, then the expression of MHC class I molecules and NKG2D ligands were assessed by flow cytometry as shown in the representative histograms from three independent experiments.
(TIF)

S6 Fig Gefitinib-induced attenuation of the expression of NKG2D ligands were reversed by Gemcitabine. A549 cells were treated with Gemcitabine (10nM) and Gefitinib (1μM) together for 24 hours and the expression of MHC class I and MICA was assessed by flow cytometry. The effects on the expressions of these molecules treated with Gemcitabine and Gefitinib together were shown as the relative MFI (rMFI) mean values of three independent experiments. Differences in means were evaluated with Student *t*-test. Bars, SEM. * -p<0.05, ** -p<0.01.
(TIF)

S7 Fig The expressions of miRs in RERF-LC-AI cells treated with Gemcitabine or A549 cell treated with Gefitinib. The RERF-LC-AI cells were treated with Gemcitabine (GEM) **(Figure A)** or A549 cells were treated with Gefitinib (GEF) for 24h **(Figure B)** then miR10b, 20a, 34c and 106 were evaluated using quantitative real-time PCR for 3 independent experiments. Differences in means were evaluated with Student *t*-test. Bars, SEM. * -p<0.05.
(TIF)

S8 Fig Transfection efficacies of miR mimic or miR inhibitor. A549 cells were transfected with 5’-fluorescein (FAM)-labeled control or miR20a mimic for 48 hours, then expression of FAM was evaluated with flow cytometry **(Figure A)**. PC–9 cells were transfected with 5’-fluorescein (FAM)-labeled LNA control or miR20a inhibitor for 24 hours, then expression of FAM was evaluated with flow cytometry **(Figure B)**.
(TIF)

S1 Table Primer for stem-loop RT-PCR and qPCR. (DOCX)

S2 Table The sequence of miR mimics and inhibitors. (DOCX)
