# Supplementary figures and images for: Contrasting Effects of the Cytotoxic Anticancer Drug Gemcitabine and the EGFR Tyrosine Kinase Inhibitor Gefitinib on NK Cell-Mediated Cytotoxicity via Regulation of NKG2D Ligand in Non-Small-Cell Lung Cancer Cells
Source: PLoS One. 2015 Oct 6;10(10):e0139809. doi: 10.1371/journal.pone.0139809 (PMC4595469; doi:10.1371/journal.pone.0139809)

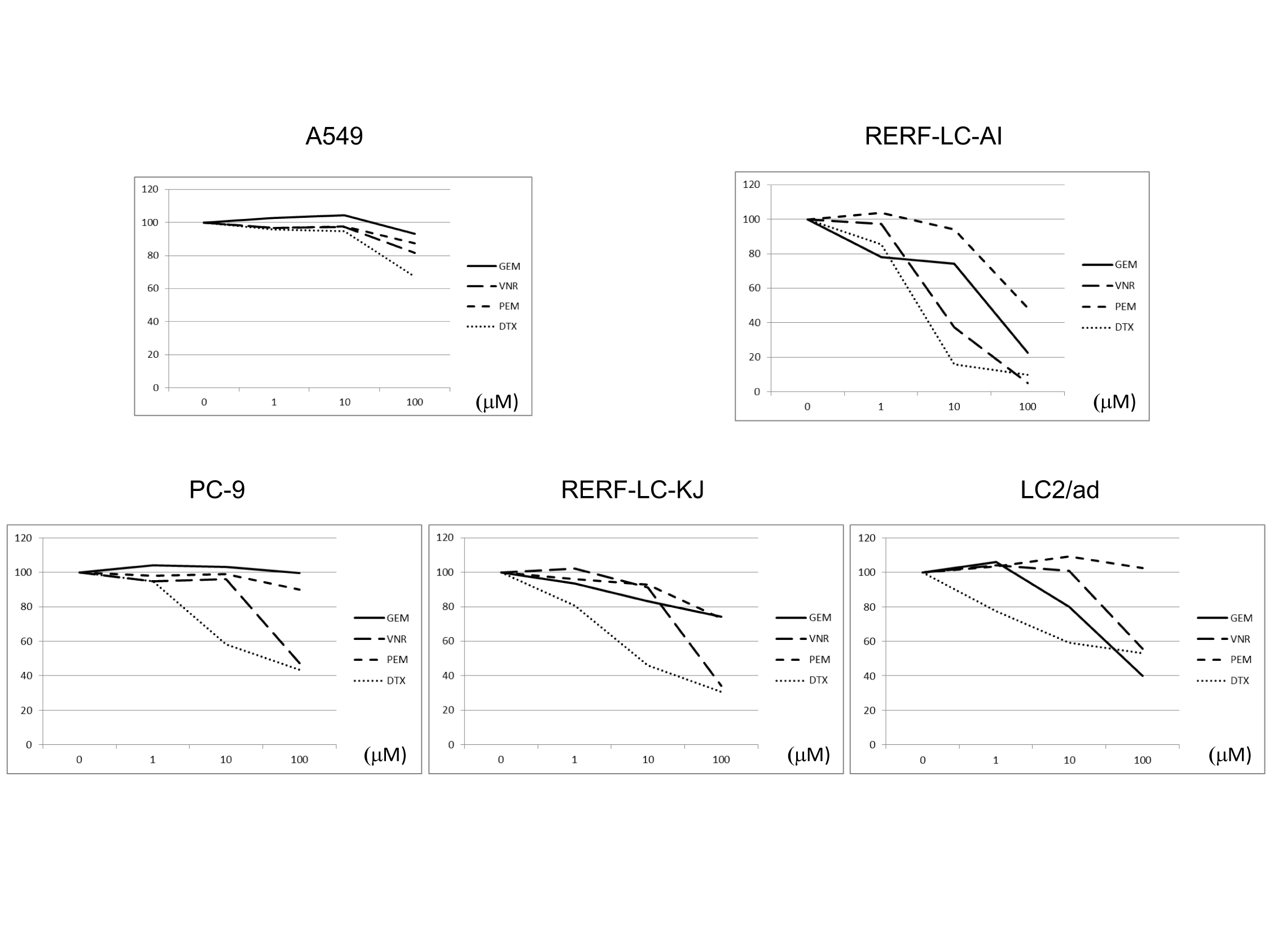

Supplement: S1 Fig — Five non-small cell lung cancer cell lines were treated with indicated concentrations of each chemotherapeutic regent for 48h. After the incubation, WST cell proliferation assay were performed. Representative data of three independent experiments are shown. (TIF) [file pone.0139809.s001.tif]

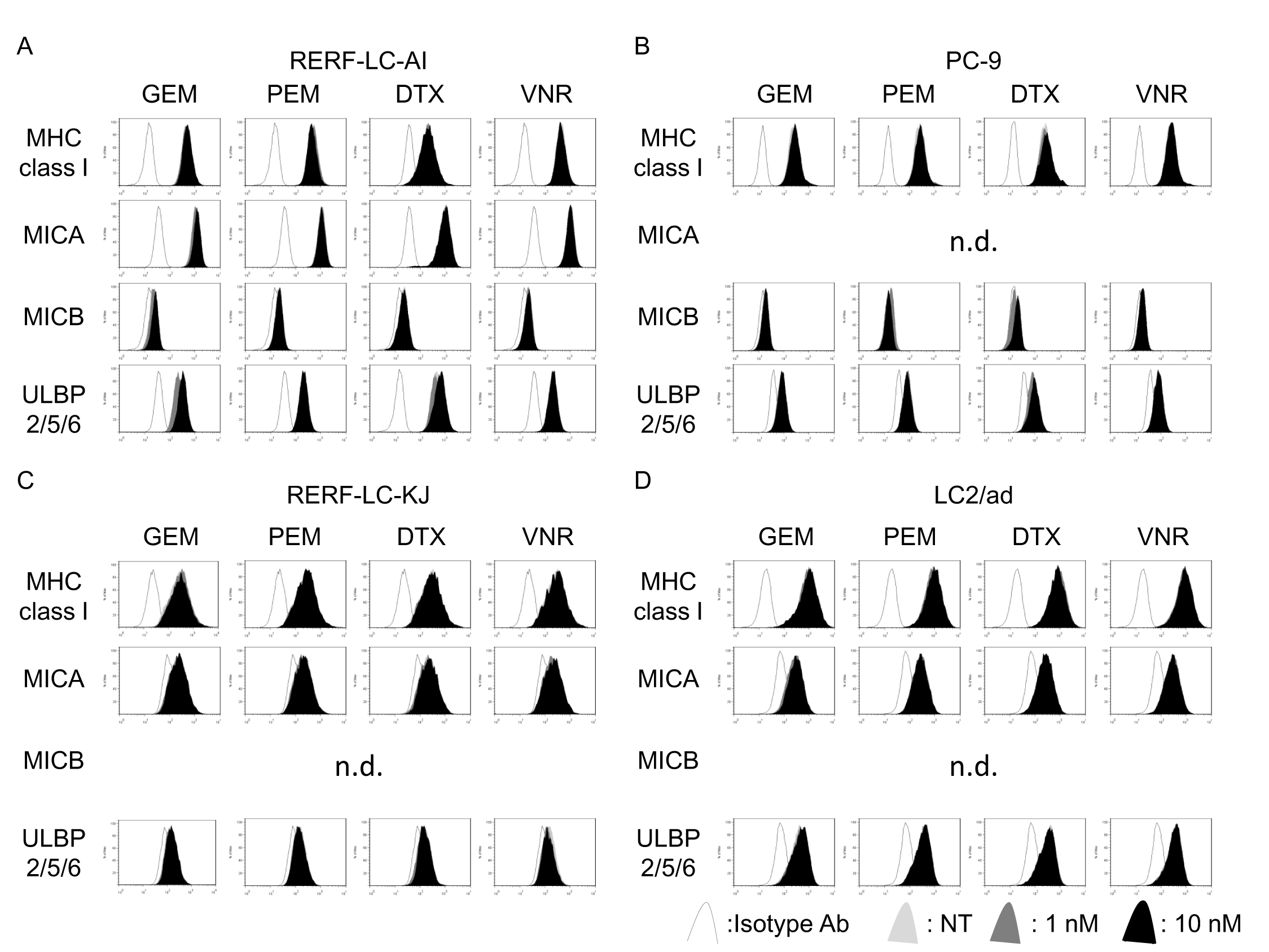

Supplement: S2 Fig — RERF-LC-AI (Figure A), PC–9 (Figure B), RERF-LC-KJ (Figure C) and LC2/ad (Figure D) cells were treated with or without 1 to 10nM of Gemcitabine (GEM), Pemetrexed (PEM), Docetaxel (DTX) or Vinorelbine (VNR) for 24 hours, then the expression of MHC class I molecules and NKG2D ligands were assessed by flow cytometry as shown in the representative histograms from three independent experiments. (TIF) [file pone.0139809.s002.tif]

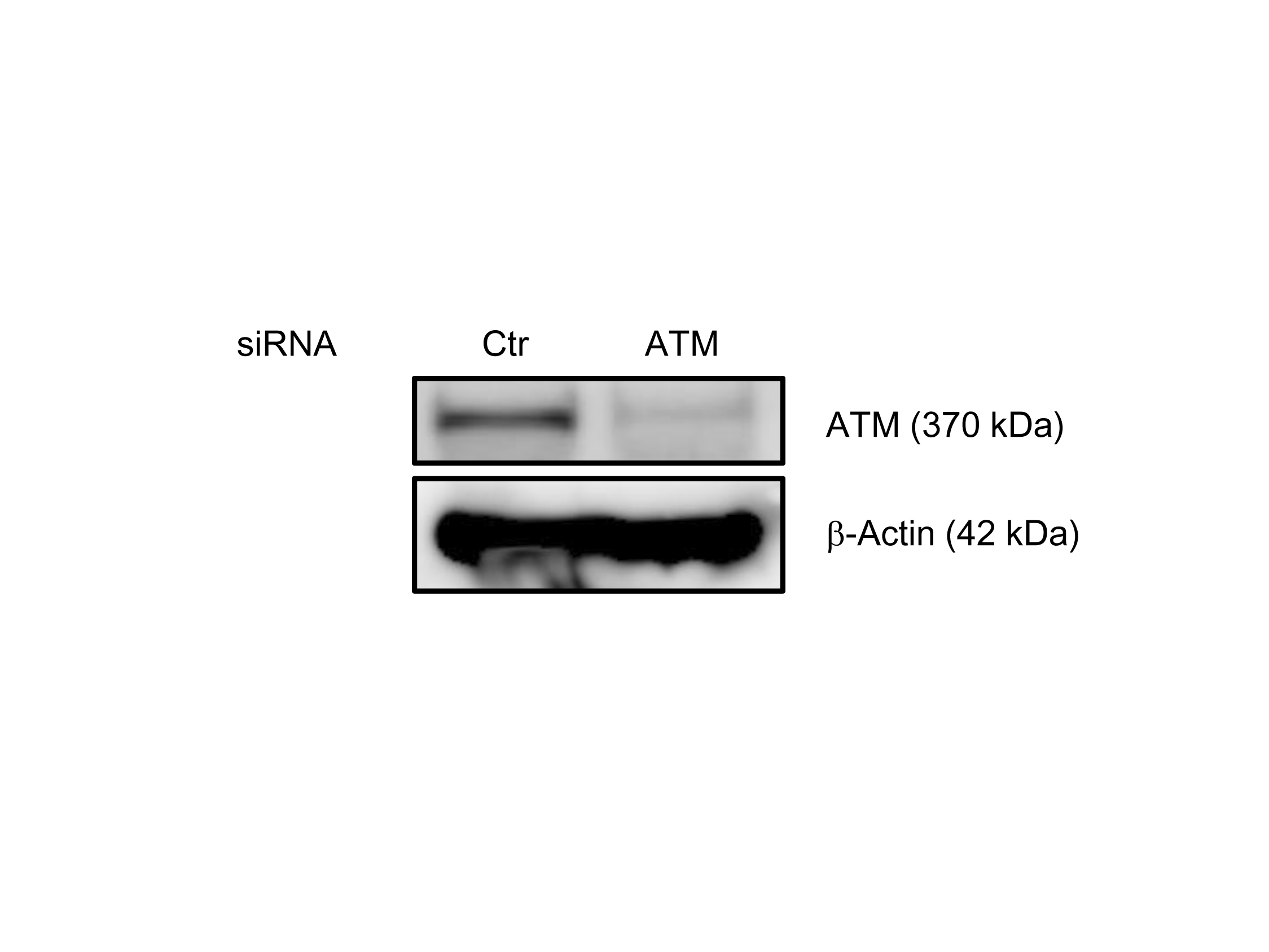

Supplement: S3 Fig — PC–9 cells were transfected with siRNA targeting ATM or control siRNA (siCtr) for 48 hours. The expression levels of ATM and β-actin were assessed by Western blot analyses. Data are presented as representatives of three independent experiments. (TIF) [file pone.0139809.s003.tif]

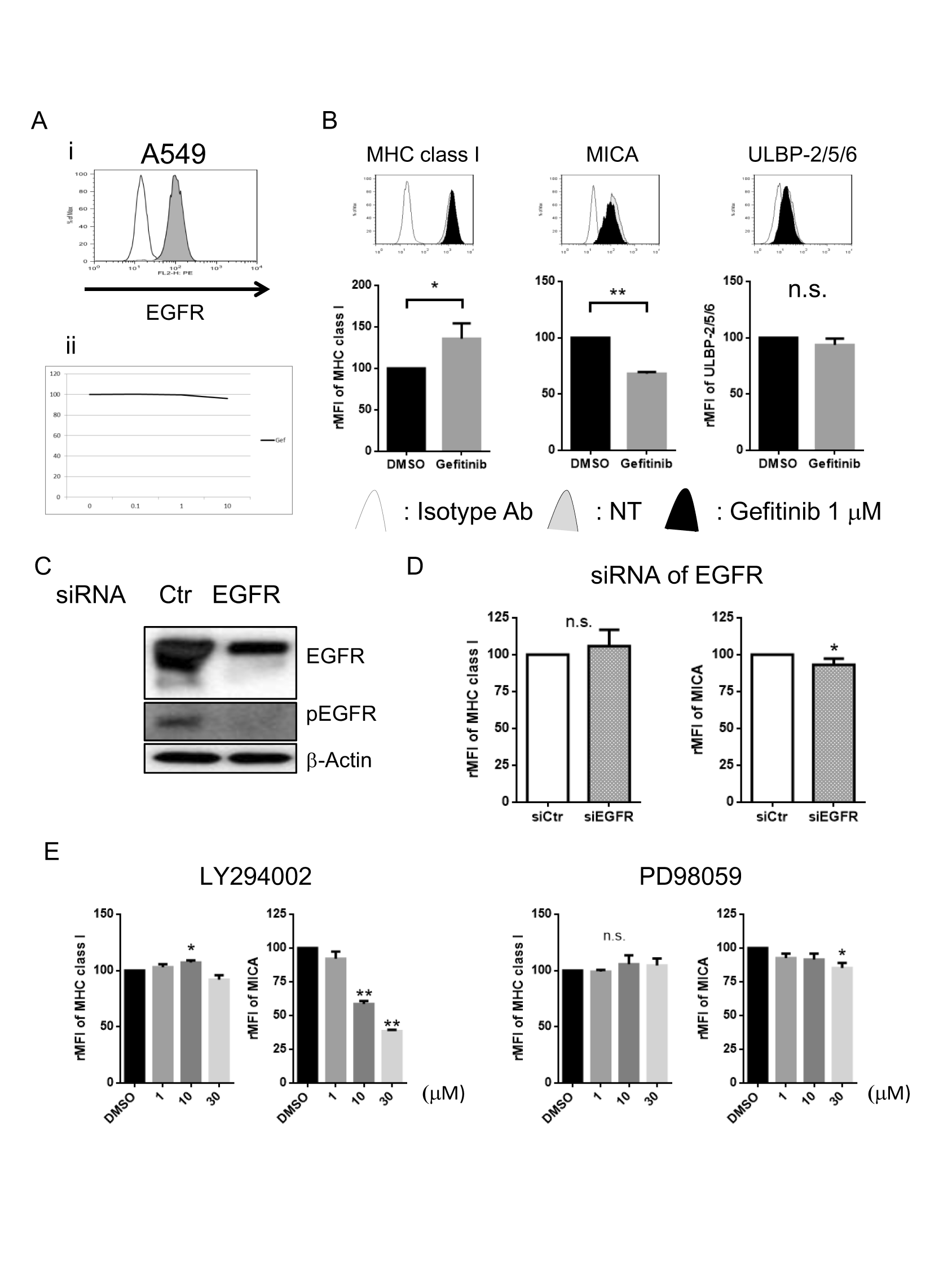

Supplement: S4 Fig — The basal expression of EGFR was assessed by flow cytometry in A549 cells (Figure Ai). WST cell proliferation assay showed A549 cells were resisitant to Gefitinib (Gef) (Figure Aii). A549 cells were treated with or without 1 μM of Gefitinib (Gef) for 24 hours. MHC class I molecules and NKG2D ligands were assessed by flow cytometry. The representative histograms from three independent experiments were shown. The relative MFI (rMFI) of MHC class I molecules, MICA, and ULBP–2/5/6 were calculated based on at least three independent experiments and evaluated with a Student t-test (Figure B). A549 cells were transfected with siRNA targeting EGFR (siEGFR) or control siRNA (siCtr) as control for 48 hours. The expressions of EGFR, phosphorylated EGFR (pEGFR) and β-actin were assessed by Western blot analyses. Data are presented as representatives of three independent experiments (Figure C). The expressions of MHC class I and MICA were assessed by flow cytometry, then the effects on the expressions of these molecules treated with siRNA of EGFR were shown as the relative MFI (rMFI) mean values of three independent experiments and evaluated with Student t-test. E: A549 cells were cultured with various concentration of the PI3K inhibitor LY294002, MEK1 inhibitor PD98059 or DMSO (0.01%) as control. The effects on the expressions of MHC class I and MICA treated with each inhibitor are shown as the relative MFI (rMFI) mean values of three independent experiments and evaluated with Student t-test (Figure D). Bars, SEM. * -p<0.05 and ** -p<0.01. (TIF) [file pone.0139809.s004.tif]

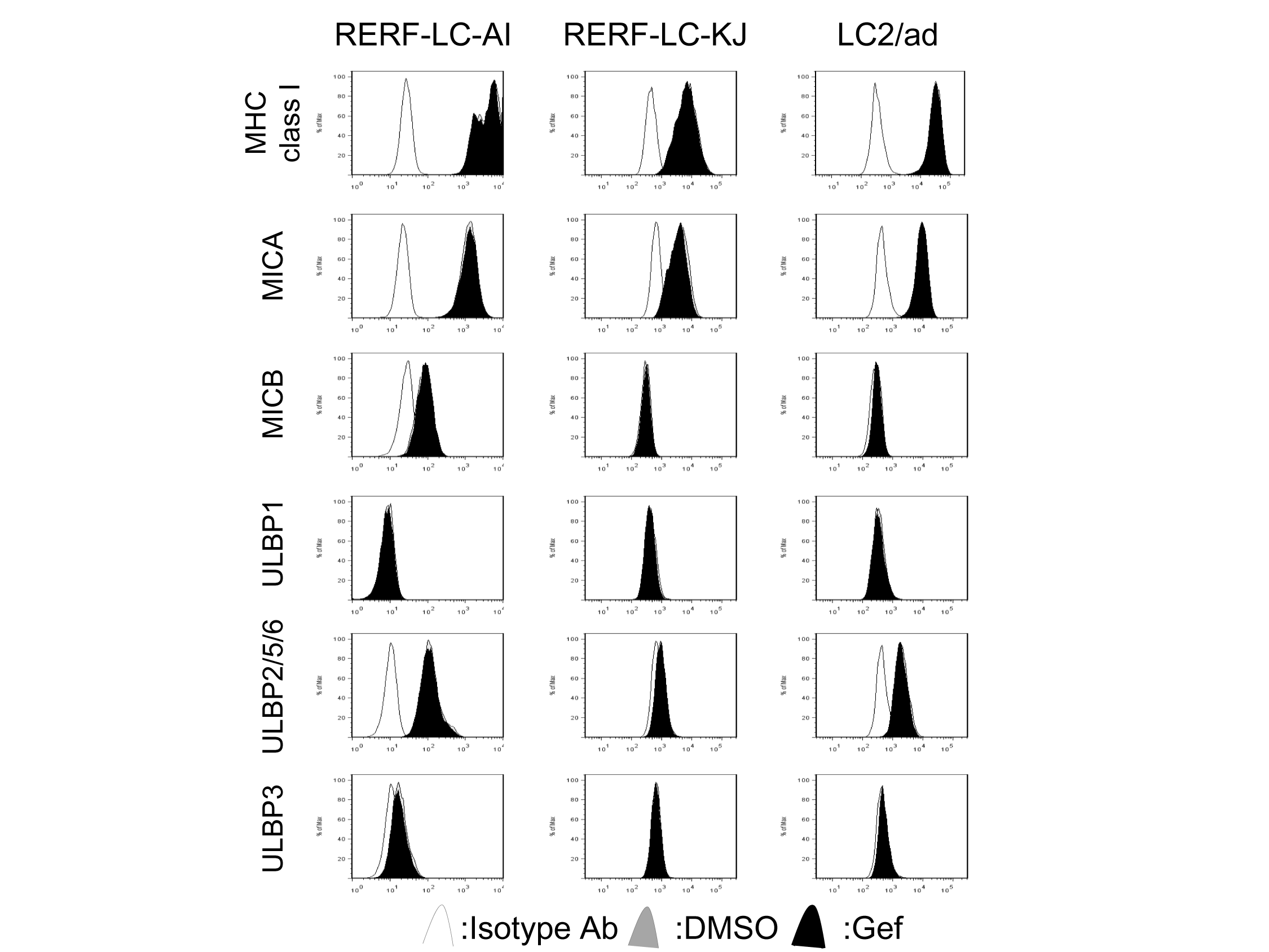

Supplement: S5 Fig — RERF-LC-AI, RERF-LC-KJ and LC2/ad cells were treated with or without 1μM of Gefitinib (Gef) for 24 hours, then the expression of MHC class I molecules and NKG2D ligands were assessed by flow cytometry as shown in the representative histograms from three independent experiments. (TIF) [file pone.0139809.s005.tif]

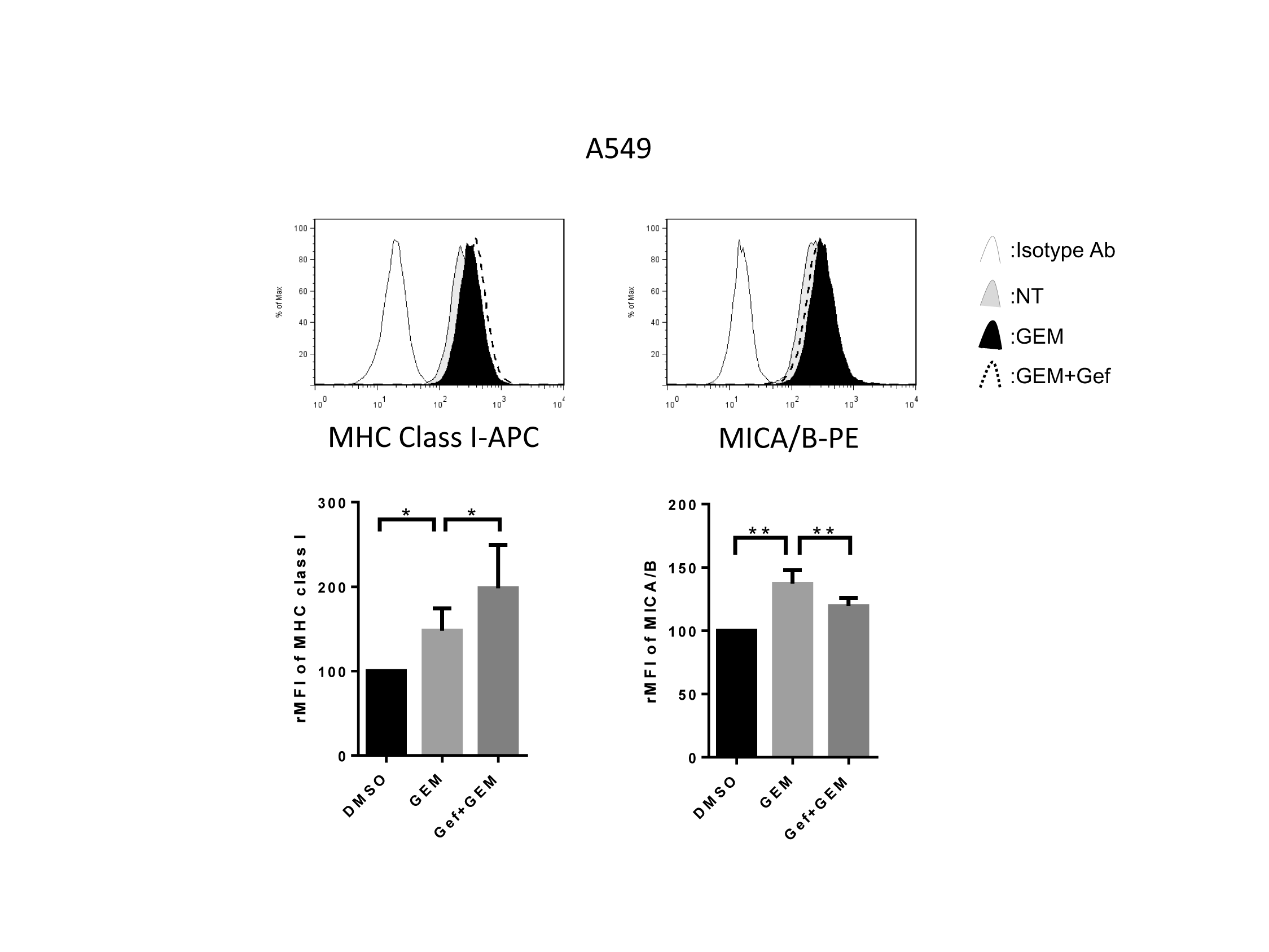

Supplement: S6 Fig — A549 cells were treated with Gemcitabine (10nM) and Gefitinib (1μM) together for 24 hours and the expression of MHC class I and MICA was assessed by flow cytometry. The effects on the expressions of these molecules treated with Gemcitabine and Gefitinib together were shown as the relative MFI (rMFI) mean values of three independent experiments. Differences in means were evaluated with Student t-test. Bars, SEM. * -p<0.05, ** -p<0.01. (TIF) [file pone.0139809.s006.tif]

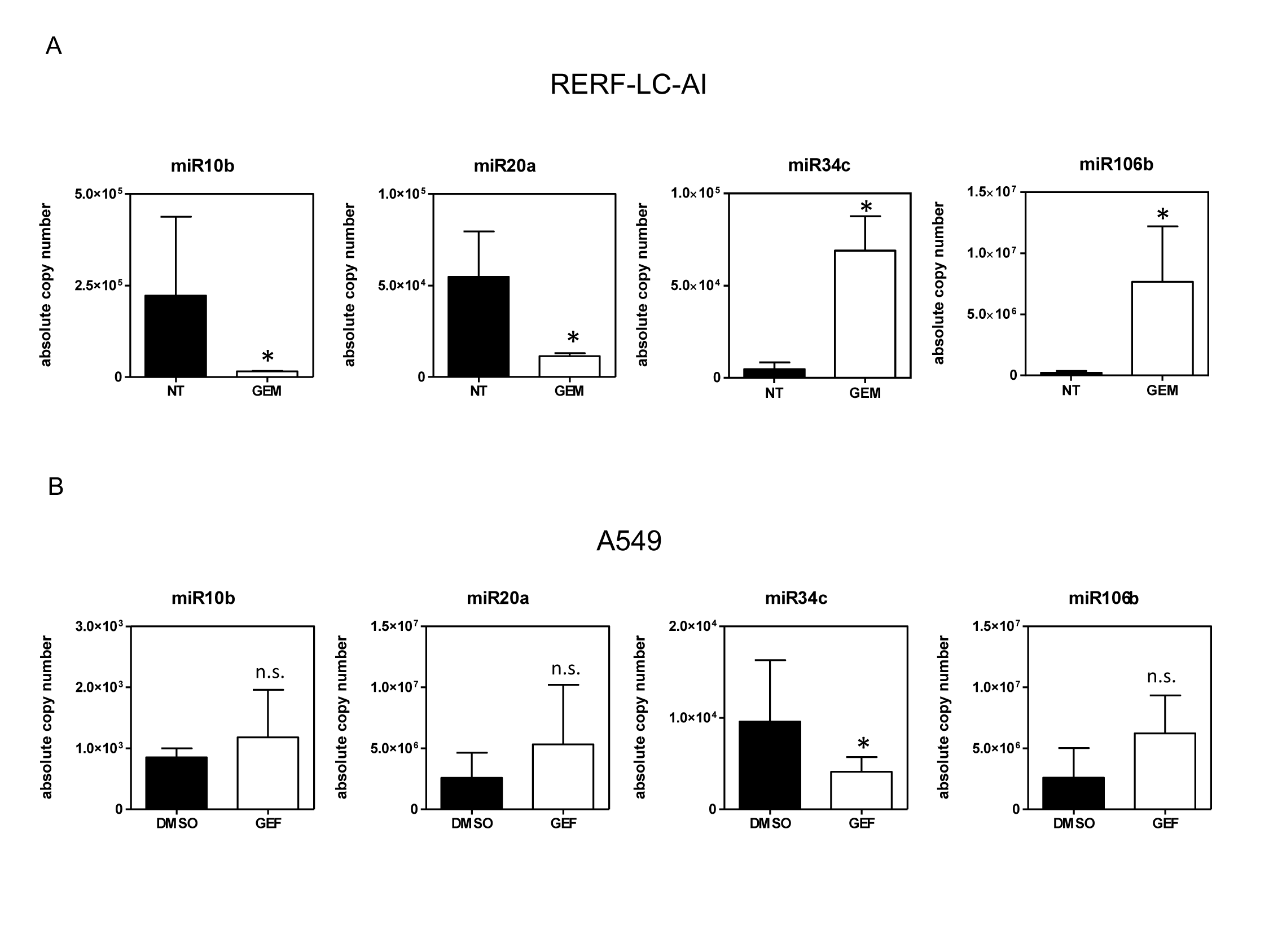

Supplement: S7 Fig — The RERF-LC-AI cells were treated with Gemcitabine (GEM) (Figure A) or A549 cells were treated with Gefitinib (GEF) for 24h (Figure B) then miR10b, 20a, 34c and 106 were evaluated using quantitative real-time PCR for 3 independent experiments. Differences in means were evaluated with Student t-test. Bars, SEM. * -p<0.05. (TIF) [file pone.0139809.s007.tif]

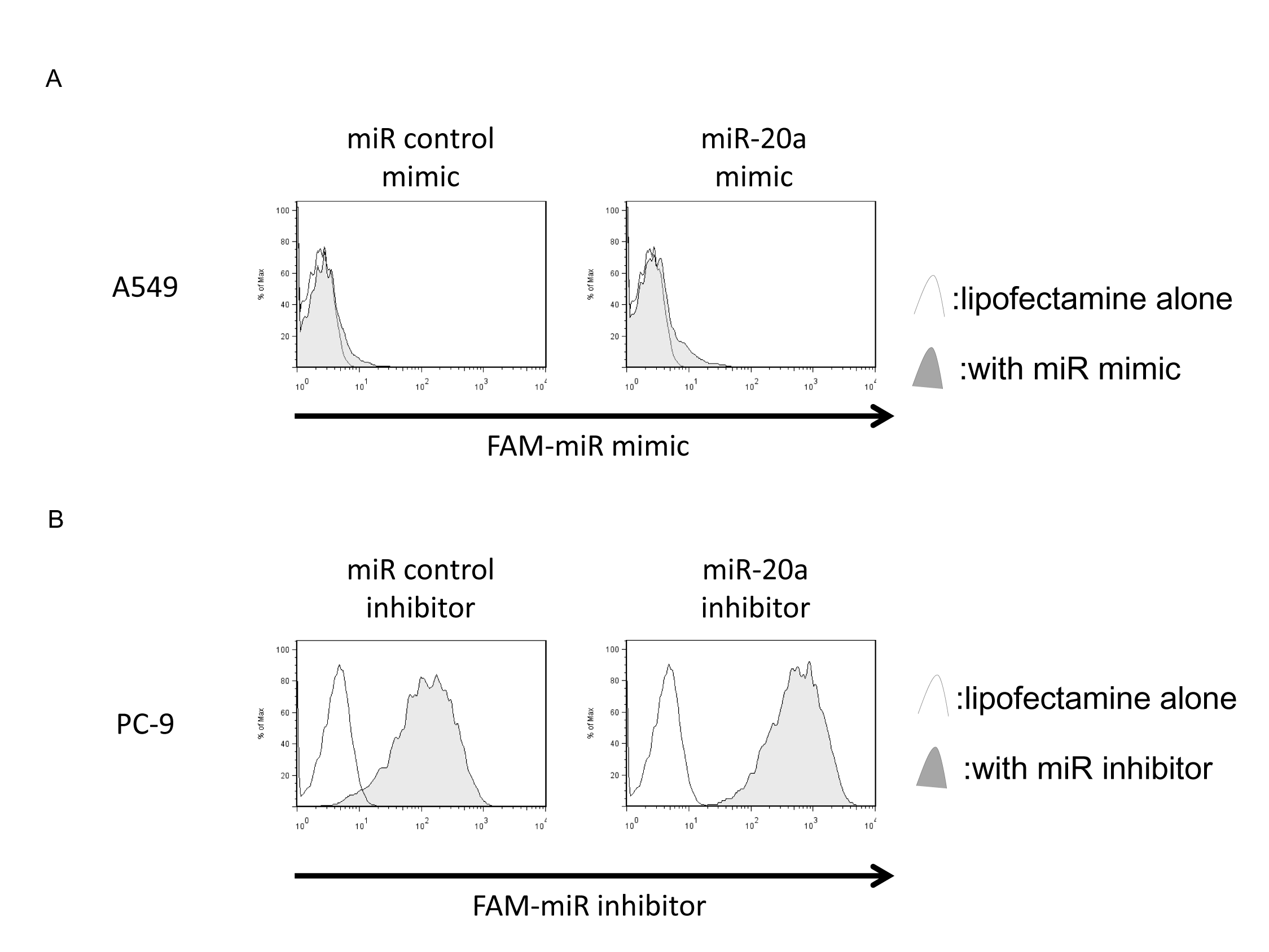

Supplement: S8 Fig — A549 cells were transfected with 5’-fluorescein (FAM)-labeled control or miR20a mimic for 48 hours, then expression of FAM was evaluated with flow cytometry (Figure A). PC–9 cells were transfected with 5’-fluorescein (FAM)-labeled LNA control or miR20a inhibitor for 24 hours, then expression of FAM was evaluated with flow cytometry (Figure B). (TIF) [file pone.0139809.s008.tif]
