# Supplementary material for: Contrasting Effects of the Cytotoxic Anticancer Drug Gemcitabine and the EGFR Tyrosine Kinase Inhibitor Gefitinib on NK Cell-Mediated Cytotoxicity via Regulation of NKG2D Ligand in Non-Small-Cell Lung Cancer Cells
Source: PLoS One. 2015 Oct 6;10(10):e0139809. doi: 10.1371/journal.pone.0139809 (PMC4595469; doi:10.1371/journal.pone.0139809)
Supplement: Supplementary file 2 [file pone.0139809.s009.docx]

miR	*stem-loop RT-PCR*	qPCR
**miR10b**	GTCGTATCCAGTGCAGGGTCCGAGGTATTCGCACTGGATACGACCACAAA	GCCCTACCCTGTAGAACCGAA
**miR20a**	GTCGTATCCAGTGCAGGGTCCGAGGTATTCGCACTGGATACGACCTACCT	GCCCTAAAGTGCTTATAGTGC
**miR34c**	GTCGTATCCAGTGCAGGGTCCGAGGTATTCGCACTGGATACGACGCAATC	GCCCAGGCAGTGTAGTTAGCT
**miR106**	GTCGTATCCAGTGCAGGGTCCGAGGTATTCGCACTGGATACGACATCTGC	GCCCTAAAGTGCTGACAGT
**miR-Rev**		GTGCAGGGTCCGAGGT
