# Supplementary material for: Contrasting Effects of the Cytotoxic Anticancer Drug Gemcitabine and the EGFR Tyrosine Kinase Inhibitor Gefitinib on NK Cell-Mediated Cytotoxicity via Regulation of NKG2D Ligand in Non-Small-Cell Lung Cancer Cells
Source: PLoS One. 2015 Oct 6;10(10):e0139809. doi: 10.1371/journal.pone.0139809 (PMC4595469; doi:10.1371/journal.pone.0139809)
Supplement: Supplementary file 3 [file pone.0139809.s010.docx]

miR-mimics/ inhibitors	Product name	Sequence
**miR-Ctr mimic**	Cel-miR-39-3p	UCACCGGGUGUAAAUCAGCUUG
**miR-20a mimic**	Has-miR-20a-3p	ACUGCAUUAUGAGCACUUAAAG
**miRCURY LNA control inhibitor**	Negative control A	TAACACGTCTATACGCCCA
**miRCURY LNA miR-20a inhibitor**	has-miR-20a-5p	ACCTGCACTATAAGCACTTTA
